# ChatGPT in precision medicine

**DOI:** 10.1063/5.0299327

**Published:** 2026-03-17

**Authors:** Chengliang Yin, Xiaochun Sun, Anlin Dai, Xin-yao Ye, Yi Lu, Wanling Wang, Yuanyuan Chen, Hong Jiang, Juan Yu, Siomui Chong, Mingming Jiang, Jiayu Xu, Bing Yang, Rajeswari Chappa, Santosh Chokkakula, Kunlun He

**Affiliations:** 1 Medical Innovation Research Department, Chinese PLA General Hospital, Beijing 100853, China; 2 National Engineering Laboratory for Medical Big Data Application Technology, Chinese PLA General Hospital, Beijing 100853, China; 3 Department of Anesthesiology, The Affiliated Traditional Chinese Medicine Hospital, Guangzhou Medical University, Guangzhou 510000, China; 4 Statistical Offices, Zhuhai People's Hospital, Zhuhai Clinical Medical College of Jinan University, Zhuhai 519000, China; 5 Department of Radiology, The First Affiliated Hospital of Shenzhen University, Health Science Center, Shenzhen Second People's Hospital, Shenzhen 518035, China; 6 Shenzhen Second People's Hospital, Shenzhen 518000, China; 7 Department of Dermatology, The First Affiliated Hospital of Jinan University and Jinan University Institute of Dermatology, Guangzhou 510630, China; 8 Medical School of Chinese PLA, Beijing 100853, China; 9 Department of Cell Biology, College of Basic Medical Sciences, Tianjin Medical University, Tianjin 300070, China; 10 Bodhan Soft Technologies, Visakhapatnam 530041, India; 11 Department of Microbiology, Chungbuk National University College of Medicine and Medical Research Institute, Cheongju, Chungbuk 28644, South Korea

## Abstract

The integration of artificial intelligence, notably the generative language model ChatGPT, into precision medicine precedes a paradigm shift in healthcare. This Review explores the capabilities of ChatGPT to process large data sets, understand contextual details, and generate human-like responses, making it a transformative tool for various applications in precision medicine. By analyzing the genetic sequences, identifying biomarkers, and predicting disease risks, ChatGPT facilitates earlier diagnosis and intervention. Moreover, its natural language processing capabilities enhance patient communication and engagement by elucidating complex medical concepts. This Review further examines ChatGPT's role in streamlining research, augmenting clinical trial recruitment, and supporting healthcare professionals through access to updated information and skill assessment. While acknowledging the complexities and potential concerns regarding reliance on publicly sourced data, this Review highlights ChatGPT's substantial capacity to optimize patient prognoses and enhance the operational efficacy of the healthcare delivery system.

## I. INTRODUCTION

Precision medicine integrates genomic, epigenomic, phenotypic, and environmental data to deliver individualized diagnostics, prognostics, and therapies, fundamentally shifting from population-based to patient-specific care paradigms.^1,2^ Explosive growth in next-generation sequencing, multi-omics profiling, and electronic health record (EHR) systems has produced heterogeneous, high-dimensional datasets that challenge traditional analytical workflows for integration and interpretation.^3–5^ Generative large language models (LLMs) like ChatGPT, leveraging advanced natural language processing (NLP), address these bottlenecks by facilitating contextual synthesis of biomedical literature, automated knowledge extraction from unstructured reports, and interpretable reasoning over complex clinical narratives.^6,7^

**FIG. 1.**
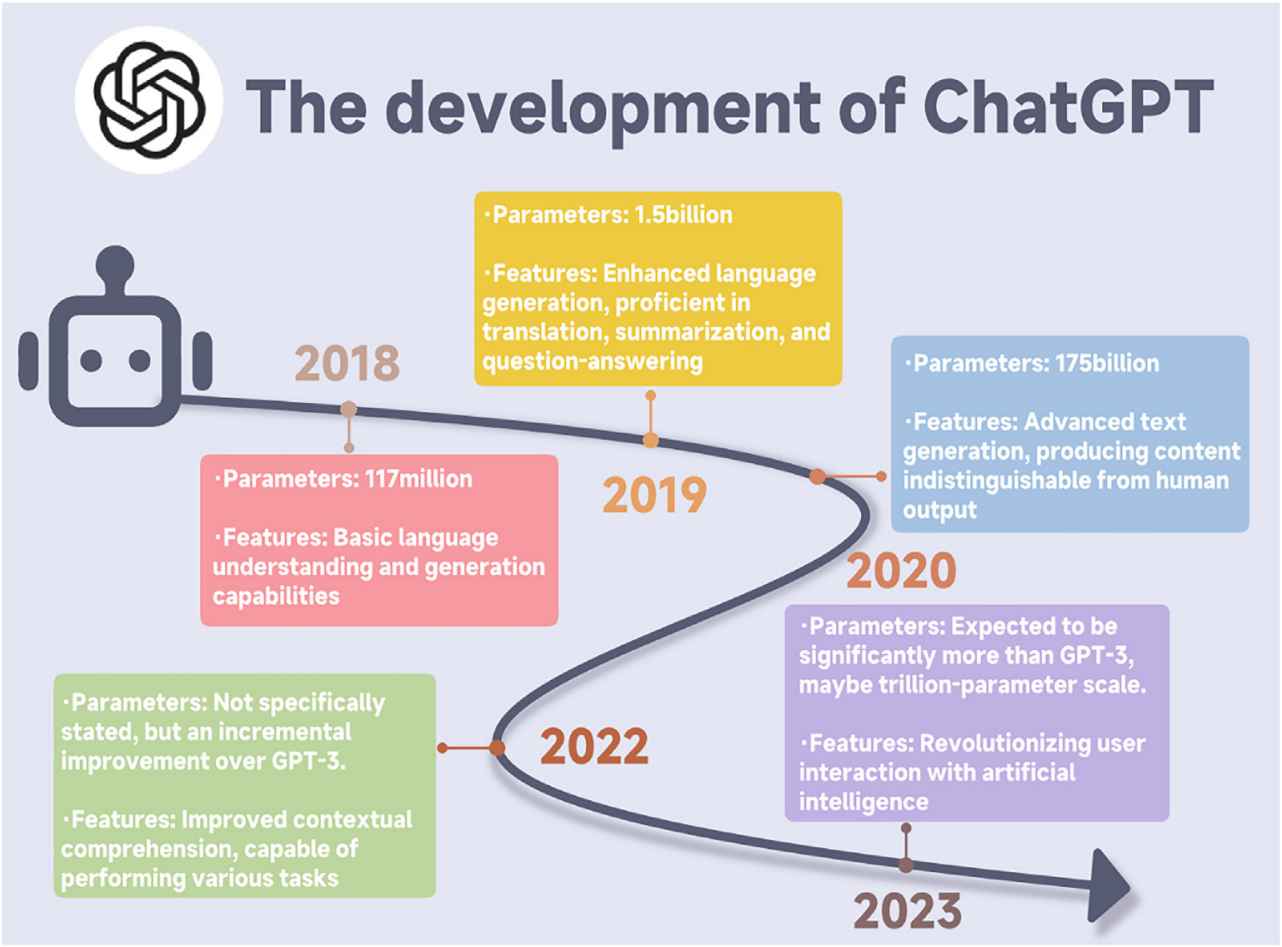
Evolution and enhancements of ChatGPT: A journey from basic language Processing to advanced interactions.

ChatGPT represents a prominent member of the LLM family, predicated on the generative pre-trained transformer (GPT) architecture.^8^ Distinct from other generative paradigms, such as generative adversarial networks (GANs) optimized for image synthesis or convolutional neural networks (CNNs) tailored to spatial pattern recognition in imaging, the GPT framework is expressly engineered for sequential text comprehension, generation, and inference. Its foundational transformer architecture harnesses multi-head self-attention mechanisms to facilitate scalable parallel computation while modeling extended contextual dependencies across token sequences, thereby surmounting the sequential bottlenecks and vanishing gradient challenges inherent to recurrent neural networks (RNNs).^9^ This architectural leap has catalyzed breakthroughs in natural language processing (NLP), equipping ChatGPT for sophisticated biomedical tasks (Fig. 1).

Contemporary iterations of ChatGPT, including GPT-3.5 and GPT-4, are developed through a multi-stage training framework comprising large-scale unsupervised pretraining on internet-scale text quantities, subsequent supervised fine-tuning on curated instruction-following datasets, and reinforcement learning from human feedback (RLHF) to improve response alignment, contextual appropriateness, and safety.^10–12^ This sequential training paradigm enables the models to generate fluent, coherent, and instruction-compliant text across a wide range of tasks. Despite these advances, the functional intelligence of ChatGPT remains fundamentally statistical and language-centric. The model operates by learning probabilistic associations among tokens and higher-order linguistic patterns rather than by encoding explicit causal relationships or mechanistic representations of biological systems. Consequently, while ChatGPT can approximate structured reasoning in textual contexts, it does not possess an intrinsic understanding of underlying molecular, cellular, or physiological processes. This limitation has important implications for its role in biomedical research and precision medicine. ChatGPT is best positioned as an interpretive and inferential support tool capable of synthesizing, contextualizing, and summarizing preprocessed biomedical information, such as variant annotations, clinical guidelines, or literature-derived knowledge. In contrast, it is not designed to perform primary computational analyses, including raw genomic data processing, molecular dynamics simulations, or quantitative pharmacokinetic and pharmacodynamic modeling, which require domain-specific algorithms and validated numerical frameworks.

In its native form, ChatGPT operates exclusively on tokenized textual inputs and lacks the capacity to directly ingest or analyze raw biomedical data formats, including genomic sequencing files, medical imaging data, or real-time clinical data streams encoded in standards such as HL7 or FHIR.^13,14^ Within precision medicine workflows, these complex data modalities must therefore undergo domain-specific preprocessing and analysis using specialized computational pipelines, after which their outputs are translated into structured or narrative representations. Once biomedical data have been transformed into interpretable textual formats, such as variant annotation reports, radiology interpretations, or curated extracts from electronic health records, ChatGPT can be applied as a secondary interpretive layer. In this role, the model can assist in synthesizing results across sources, contextualizing findings within existing knowledge, and generating explanatory narratives that support clinical communication, documentation, and education. A representative application is pharmacogenomics, which investigates how genetic variation influences drug efficacy and toxicity.^15,16^ In this setting, ChatGPT may facilitate the summarization of gene–drug associations from curated knowledge bases, assist in interpreting guideline-based recommendations, and help translate technical pharmacogenomic test results into patient-oriented explanations. Importantly, such contributions are contingent on the accuracy and completeness of upstream analyses and should be viewed as augmentative rather than substitutive to expert-driven interpretation.

Empirical evaluations demonstrate that ChatGPT-3 and GPT-4 achieve competitive performance on biomedical question-answering tasks and clinical reasoning benchmarks, including support for knowledge retrieval, medical licensing exam preparation, and hypothesis formulation.^17–20^ However, accuracy diminishes substantially when processing multimodal inputs or noisy real-world datasets prevalent in precision medicine workflows involving heterogeneous omics, imaging, and EHR data. These limitations underscore the imperative for domain-specific fine-tuning, retrieval augmentation, and prospective clinical validation prior to deployment in decision-support applications.

Despite substantial capabilities, persistent challenges remain. ChatGPT is prone to hallucinations, generating linguistically coherent yet factually erroneous outputs, and may propagate biases, outdated practices, or underrepresented perspectives embedded in its pretraining corpora.^21–23^ These vulnerabilities threaten patient safety and health equity in precision medicine contexts, where erroneous genomic interpretations or biased pharmacogenomic recommendations could exacerbate disparities. Mitigation strategies encompass retrieval-augmented generation (RAG) to anchor responses in verified biomedical corpora, tool-augmented reasoning interfacing with curated databases (ClinVar, PharmGKB, OMIM), and grounded prompting enforcing evidence citation alongside post-hoc fact-checking pipelines.^24–28^ Integration of these safeguards demonstrably improves factual accuracy and calibration, though prospective clinical validation and governance frameworks remain indispensable for safe deployment.

**FIG. 2.**
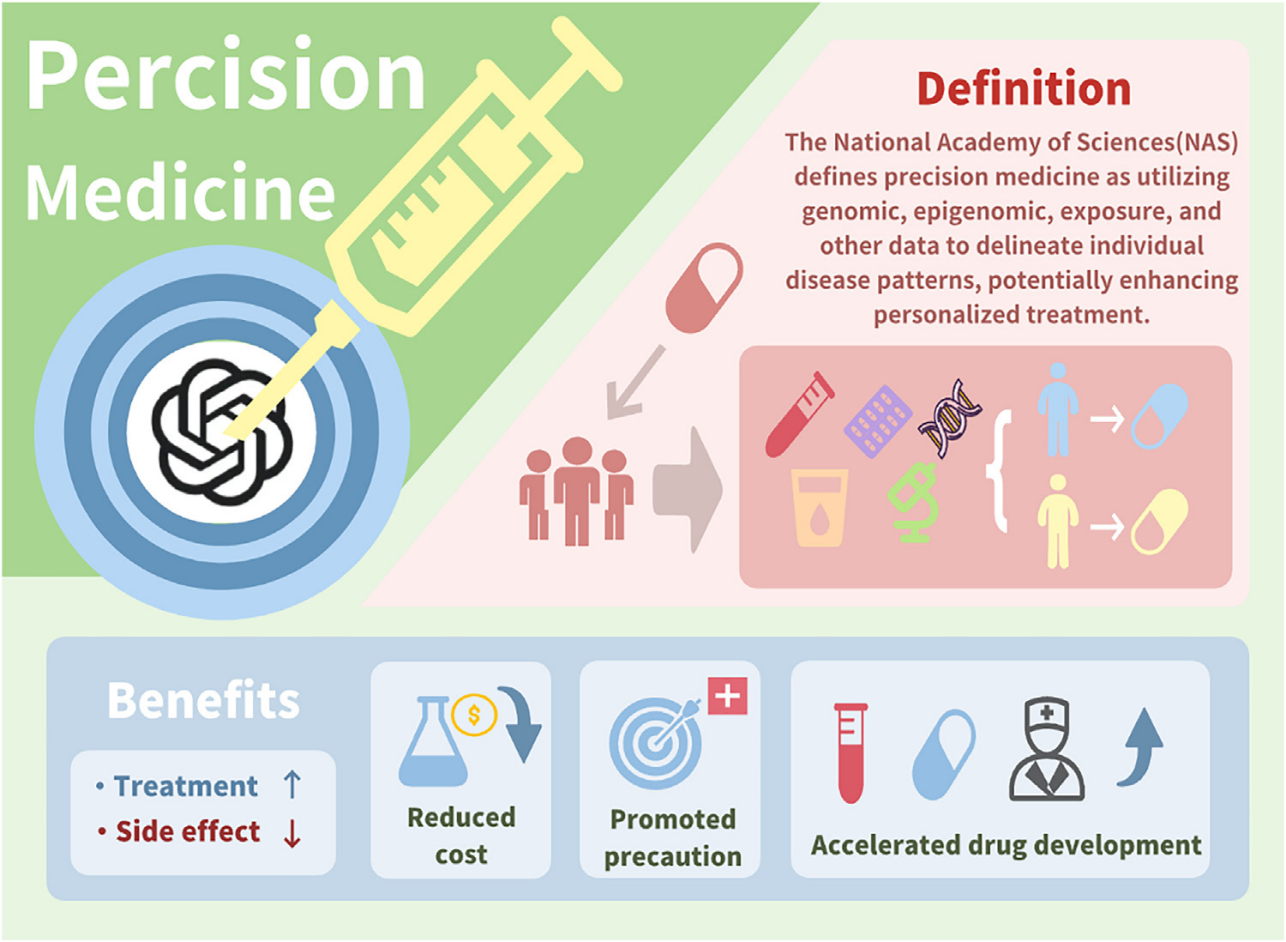
The definition and advantages of precision medicine.

Given the transformative abilities of both ChatGPT and precision medicine, this review examines how ChatGPT can optimize various aspects of precision medicine, including data analysis, patient communication, diagnosis, decision-making, disease prediction analysis, and remote patient monitoring (Fig. 2). While acknowledging the challenges associated with using publicly sourced data, this review highlights the substantial potential of ChatGPT in advancing personalized and precise medical practices, ultimately improving patient outcomes and healthcare efficiency.

We performed a comprehensive literature search using PubMed, Web of Science, Scopus, and Google Scholar. The search strategy combined keywords such as “ChatGPT,” “large language models,” “artificial intelligence,” “precision medicine,” “personalized medicine,” “drug discovery,” “diagnosis,” “clinical decision support,” and “patient education,” using Boolean operators to refine results. Reference lists of retrieved publications were also screened to identify additional relevant studies.

Articles were considered eligible if they discussed ChatGPT or related large language models in the context of healthcare and precision medicine. We included original research papers, reviews, case studies, preprints, and authoritative reports published in English that described applications in areas such as diagnosis, prognosis, drug discovery, personalized treatment, patient communication, or healthcare delivery. Studies were excluded if they were unrelated to medicine, published in languages other than English, or focused solely on technical aspects of AI without clinical or biomedical relevance.

From each study, we extracted information regarding objectives, clinical domains, applications of ChatGPT or other large language models, reported outcomes, and limitations. Because of the heterogeneity in study designs and outcomes, a meta-analysis was not feasible. Instead, findings were synthesized narratively and organized thematically into key domains, including precision diagnosis, decision-making and clinical support, drug discovery and personalized treatment, multi-omics data integration, patient communication and education, predictive analytics and prevention, remote monitoring and chronic disease management, as well as opportunities, challenges, and future perspectives.

## II. ChatGPT IN PRECISION DIAGNOSIS

The rapid evolution of LLMs, exemplified by ChatGPT, has catalyzed a paradigm shift in artificial intelligence applications within precision diagnosis.^29–31^ Across an expanding body of empirical research, LLMs have been systematically evaluated in diverse diagnostic contexts, including symptom-based clinical reasoning, differential diagnosis generation, clinical vignette interpretation, triage prioritization, and patient-specific risk stratification.^32–34^ Collectively, these investigations demonstrate that LLMs can approximate clinician-level diagnostic performance in constrained, well-structured tasks, particularly when operating on standardized prompts or clearly defined clinical scenarios, while exhibiting marked performance variability in open-ended or high-uncertainty diagnostic contexts.^35–37^ This accumulating evidence-based positions ChatGPT not as an autonomous diagnostic authority, but rather as a probabilistic reasoning engine with the potential to augment clinical cognition and decision-making processes.^38,39^

Clinical diagnosis represents a fundamentally distinct cognitive domain from conventional algorithmic pattern recognition tasks. It necessitates longitudinal integration of heterogeneous data streams, including symptom narratives, evolving laboratory values, imaging findings, comorbidity profiles, medication histories, and contextual modifiers under inherent conditions of epistemic uncertainty.^40^ Unlike radiologic or histopathologic diagnostics, where artificial intelligence performance gains are predominantly driven by supervised learning on large-scale annotated image datasets, clinical diagnostic reasoning depends critically on narrative synthesis, semantic understanding, and iterative hypothesis refinement.^41,42^ Multiple published evaluations indicate that LLMs demonstrate particular efficacy in this domain, exhibiting strengths in organizing unstructured electronic health record (EHR) data, generating ranked differential diagnoses with associated probability estimates, and identifying atypical or contradictory clinical features that may be overlooked during cognitively demanding clinical encounters or in high patient-volume settings.^43,44^

Epidemiological analyses of diagnostic error consistently demonstrate that patient harm most frequently arises from missed or delayed recognition of common, high-prevalence conditions rather than failure to identify rare diagnoses. In response to this well-documented pattern, recent empirical studies suggest that LLMs may function as cognitive decision-support systems by systematically reinforcing consideration of prevalent diagnoses, guideline-concordant diagnostic pathways, and established risk factor disease associations.^45^ When integrated into EHR-driven clinical workflows, LLMs have demonstrated the capacity to rapidly synthesize extensive longitudinal patient records, encompassing clinician notes, laboratory time series, imaging report summaries, prior diagnoses, and social determinants of health into coherent, clinically interpretable narratives.^46^ This synthesis capability addresses a task that frequently exceeds human cognitive bandwidth constraints in contemporary practice environments characterized by information overload and time pressure.

Nevertheless, the published literature simultaneously highlights critical limitations that constrain immediate clinical deployment.^47^ Diagnostic accuracy exhibits high sensitivity to prompt engineering, data completeness, input formatting, and task framing.^48^ LLMs remain vulnerable to generating hallucinated information, overconfident probability estimates, and internally inconsistent outputs, particularly in cases involving ambiguous presentations, conflicting data sources, or rare disease entities with limited representation in training corpora.^21,49^ Furthermore, these models lack explicit mechanisms for uncertainty quantification, causal reasoning, and temporal pattern recognition, cognitive capabilities essential for robust clinical diagnosis. Consequently, current evidence supports the judicious use of ChatGPT as an assistive diagnostic layer that enhances information synthesis, hypothesis generation, and cognitive debiasing, rather than as an independent or replacement diagnostic system.

Ongoing research increasingly emphasizes the imperative for hybrid diagnostic architectures that strategically combine general-purpose LLMs with domain-specific disease models, explicit Bayesian uncertainty quantification frameworks, structured clinical knowledge graphs, and continuous clinician oversight mechanisms.^50,51^ Such integrated systems would preserve the semantic flexibility and data synthesis capabilities of LLMs while mitigating their known failure modes through complementary computational approaches and human-in-the-loop validation. Future development trajectories must prioritize rigorous prospective validation in real-world clinical environments, transparent reporting of model limitations and failure modes, establishment of clear accountability frameworks, and continuous monitoring for performance drift, bias propagation, and unintended consequences.^52^ Only through such systematic, evidence-based implementation can LLMs fulfill their potential to enhance diagnostic precision while maintaining patient safety, clinical accountability, and professional standards of care.

Thus, ChatGPT has proven highly effective in significantly improving diagnostic accuracy across a wide range of crucial domains, including cancer, cardiovascular and cerebrovascular disorders, HIV/AIDS, diabetes, and rare diseases.

### A. Cancer

**FIG. 3.**
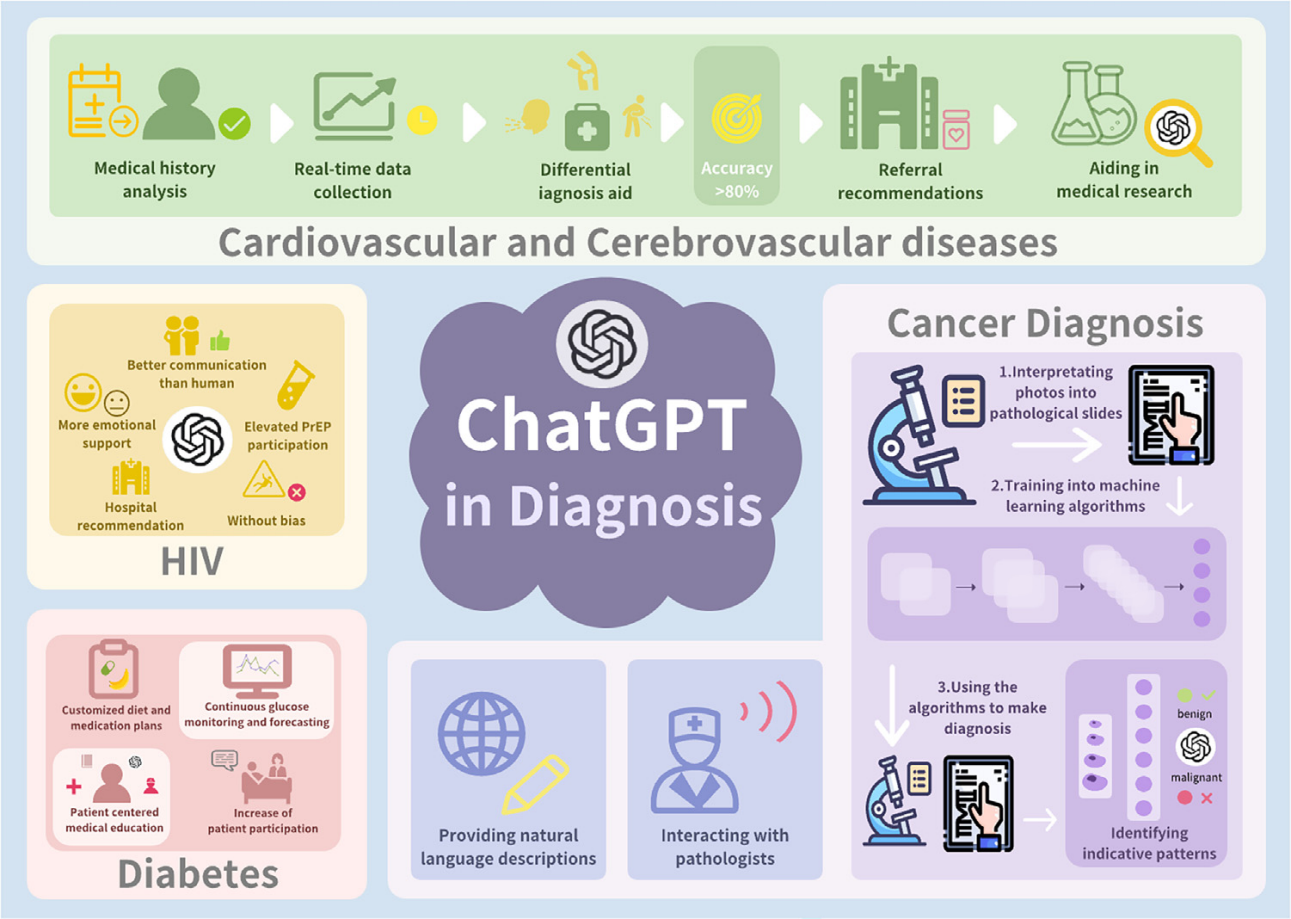
Comprehensive application of ChatGPT in disease diagnosis and treatment, with a focus on cancer pathology, cardiovascular and cerebrovascular diagnostics, and HIV/AIDS patient services.

Accurate cancer diagnosis critically determines therapeutic efficacy, survival, and quality of life. Tumorigenesis typically follows a prolonged, heterogeneous trajectory, with early-stage malignancies frequently presenting nonspecific symptoms that challenge timely detection.^2^ Comprehensive oncological diagnosis necessitates systematic integration of multimodal data, including detailed medical histories, imaging studies, histopathological examination, immunohistochemical profiling, and molecular genetic characterization (Fig. 3).^1^ Large language models such as ChatGPT may function as adjunctive tools within this diagnostic workflow by facilitating information synthesis and augmenting clinical reasoning.^53^

Comprehensive medical history-taking remains foundational to cancer diagnosis, yet temporal constraints and disease heterogeneity frequently compromise data completeness. ChatGPT's natural language processing enables adaptive, structured questioning that may enhance systematic elicitation of clinically relevant information, including symptom chronology, familial cancer predisposition, environmental exposures, and lifestyle risk factors often inadequately captured during routine encounters.^54^ When appropriately instructed, ChatGPT can extract salient elements from unstructured dialogue and transform them into standardized electronic medical record entries, facilitating interdisciplinary communication and diagnostic continuity. Nevertheless, prompt-dependent variability and occasional omissions necessitate direct clinician oversight and verification.^55–57^

ChatGPT demonstrates variable but measurable diagnostic utility across cancer types (Table I). Evidence indicates relatively high accuracy for patient-oriented inquiries in head and neck, prostate, colorectal, pancreatic, and lung malignancies, particularly for screening logic, symptom interpretation, and guideline-concordant diagnostic pathways. Performance proves less reliable for complex decision-making in hepatocellular carcinoma and cervical cancer, where incomplete surveillance recommendations, outdated criteria, or insufficient treatment-linked reasoning have been documented.^58,59^ These findings indicate ChatGPT performs optimally as a support tool for initial evaluation and patient education rather than as an autonomous diagnostic system.

**TABLE I.** ChatGPT in the precision diagnosis of cancer.

Cancer Type	Diagnostic Task Evaluated	Performance in Diagnosis	Key Diagnostic Limitations
Head and neck	Patient questions on diagnosis, staging, and workup	High diagnostic accuracy: 86.4% responses rated complete/correct; strong reproducibility	Knowledge cutoff; lower reliability than Google for some diagnostic queries
Prostate	Screening and diagnostic interpretation (PSA, biopsy indication)	High accuracy (>90%); clear explanation that PSA alone is insufficient; appropriate referral advice	Reduced accuracy in scenario-based or guideline-specific diagnostic decisions
Hepatocellular Carcinoma	Surveillance and diagnostic pathways	Correct identification of cirrhosis as an indication for surveillance; reasonable diagnostic explanations	Incomplete diagnostic pathways; omission of CT/MRI in specific settings; outdated criteria
Breast	Diagnosis of implant-associated malignancies (BIA-ALCL)	Accurate and high-quality diagnostic explanations; superior to Google Bard	Limited to rare, implant-specific diagnostic contexts
Lung	Screening eligibility and radiologic terminology	Higher proportion of correct diagnostic answers than Bard and Bing; good explanation of imaging terms	∼18% incorrect responses; inconsistent screening recommendations
Colon	Diagnostic approach and guideline-based workup	86.8% diagnostic answers rated appropriate: high concordance with ASCRS guidance	Limited depth for complex diagnostic scenarios
Pancreatic	Diagnostic process and pre-surgical assessment	The majority of diagnostic responses were rated very good/excellent by experts	Limited personalization; weak handling of borderline resectability
Cervical	Screening, diagnosis, and staging	Only 53.1% of diagnostic answers were rated correct and comprehensive	Poor performance in treatment-linked diagnostic decision pathways

Radiological imaging remains indispensable for tumor detection, staging, and monitoring. Although ChatGPT cannot directly analyze raw imaging data, studies demonstrate its capacity to synthesize structured radiology reports, integrate imaging findings with clinical context, and identify diagnostic gaps warranting investigation.^60^ Additionally, ChatGPT can translate specialized radiological terminology into patient-accessible language, potentially enhancing health literacy and engagement during diagnostic evaluation. However, documented inaccuracies and oversimplification necessitate strict positioning of LLMs as supplementary tools, with final diagnostic authority residing with board-certified radiologists.^61^

Pathological diagnosis, encompassing histomorphological assessment, immunohistochemical profiling, and molecular genetic testing, has become central to tumor classification and precision oncology.^62^ When provided structured input, ChatGPT can assist in synthesizing immunohistochemical marker patterns, correlating immunophenotypes with tumor subtypes, and contextualizing findings within established diagnostic frameworks.^63^ Furthermore, ChatGPT may support mapping molecular alterations, including somatic mutations, gene amplifications, and chromosomal rearrangements, to evidence-based therapeutic options, particularly in cases involving multiple actionable mutations or acquired resistance. However, the rapidly evolving landscape of oncological biomarkers and guidelines mandates continuous model updating and mandatory expert review.

By integrating longitudinal clinical histories, imaging summaries, histopathological descriptions, immunohistochemical profiles, and molecular genetic results, ChatGPT offers a framework for supporting multimodal diagnostic reasoning in oncology.^64^ Such synthesis may enhance diagnostic efficiency, reduce information fragmentation, and improve adherence to evidence-based algorithms, particularly in resource-limited settings. However, critical limitations, including a propensity for hallucinated information, dependency on input data quality, the absence of uncertainty quantification mechanisms, and an inability to assume clinical accountability, necessitate cautious implementation.^23^

ChatGPT should be conceptualized strictly as a clinician-supervised decision-support system augmenting human expertise rather than an autonomous diagnostic tool. Optimal integration requires prospective validation in diverse clinical environments, robust governance frameworks delineating appropriate use cases, transparent communication of system limitations to clinicians and patients, continuous performance monitoring for accuracy drift and bias, and adherence to regulatory standards.^65^ Only through rigorous, evidence-based implementation can LLMs augment oncological diagnosis while preserving patient safety, clinical accountability, and diagnostic integrity.

### B. Cardiovascular and cerebrovascular disorders

Cardiovascular and cerebrovascular diseases arise from complex, multifactorial pathophysiological processes involving genetic predisposition, environmental determinants, and modifiable behavioral risk factors.^66,67^ Accurate diagnosis necessitates rapid integration of patient-reported symptoms, clinical history, physical examination findings, laboratory biomarkers, and imaging data.^68^ Diagnostic delay or misclassification remains prevalent, particularly in atypical presentations of acute coronary syndrome (ACS), heart failure, and ischemic stroke, highlighting the need for decision-support tools capable of augmenting early diagnostic reasoning (Fig. 3).^69^

One of ChatGPT's most consistently demonstrated diagnostic capabilities is its capacity to interpret unstructured, patient-reported symptoms expressed in natural language.^70^ Evaluation using ten real-world cardiovascular case scenarios reported correct identification of the primary diagnosis in eight cases, with the correct diagnosis included within the differential diagnosis in the remaining two cases.^71^ Notably, this study demonstrated that ChatGPT exhibited particular proficiency in recognizing classical symptom patterns associated with ischemic heart disease, cardiomyopathy, and valvular disorders.

In neurological contexts, examination of ChatGPT's ability to respond to cardiopulmonary resuscitation and stroke-related symptom descriptions found that the model consistently recognized red-flag neurological features, including unilateral weakness, speech disturbance, and sudden loss of consciousness, frequently recommending immediate emergency evaluation.^72^ Although not designed as a formal diagnostic accuracy study, these findings support ChatGPT's potential utility in early triage and symptom recognition.

Cross-sectional evaluation using 100 higher-order clinical reasoning questions across internal medicine subspecialties, including cardiology and cerebrovascular disease, reported an overall diagnostic accuracy of approximately 75%.^73^ Performance was highest for symptom-based reasoning and lowest for multi-step diagnostic synthesis requiring integration of longitudinal laboratory or imaging data, underscoring the model's current limitations in complex diagnostic workflows.

Several investigations have examined ChatGPT's ability to generate differential diagnoses consistent with contemporary clinical practice guidelines.^74^ Evaluation of performance using questions from the European Examination in Core Cardiology found that the model achieved a correct response rate of approximately 60%, meeting the passing threshold.^75^ The model performed optimally for ischemic heart disease and cardiac arrhythmias, while diagnostic accuracy was diminished for advanced heart failure and congenital heart disease.

Similarly, assessment of ChatGPT's performance on the United States Medical Licensing Examination (USMLE), which encompasses substantial cardiovascular content, reported that the model performed at or near the passing threshold.^17^ While ChatGPT demonstrated competency in pattern recognition and guideline-based clinical associations, it exhibited difficulty with questions requiring nuanced interpretation of diagnostic test results, reinforcing its role as a generalist-level diagnostic support tool rather than a subspecialist-equivalent system.

Evaluation of ChatGPT's responses to clinical questions derived from the Japanese Society of Hypertension Guidelines demonstrated high overall accuracy, although performance varied depending on the strength and clarity of available supporting evidence.^76^ This variability reflects ChatGPT's fundamental dependence on the quality and representativeness of its training data and highlights the risk of inconsistent recommendations in clinical areas characterized by evolving or conflicting evidence.

Risk stratification constitutes a central component of cardiovascular and cerebrovascular diagnosis, particularly for primary and secondary prevention strategies.^77^ Although ChatGPT does not natively calculate validated clinical risk scores, studies suggest it can appropriately synthesize traditional cardiovascular risk factors when explicitly prompted.^78^ In preventive cardiology simulations, ChatGPT-generated recommendations aligned broadly with established frameworks such as SCORE2 and Framingham risk assessment concepts, although investigators consistently emphasize caution against unsupervised clinical application.

One study demonstrated that ChatGPT provided guideline-concordant preventive recommendations in 22 of 25 scenarios within a breast cancer prevention context.^79^ Subsequent cardiovascular-focused applications employing similar methodology reported comparable performance when addressing statin therapy initiation, blood pressure treatment targets, and lifestyle modification interventions. However, these studies also emphasized the risk of inappropriate oversimplification when patient-specific modifying factors were not explicitly incorporated.

The diagnostic implications of ChatGPT in telemedicine and patient-facing applications have also been investigated.^80^ Comparison of ChatGPT-generated patient education materials with expert-authored content found that while ChatGPT produced content rapidly, accuracy and readability were inferior to materials generated by clinical experts. Nonetheless, the accessibility and scalability of ChatGPT suggest potential utility for preliminary symptom assessment and patient engagement, particularly in medically underserved or resource-constrained settings.

Several studies evaluating conversational artificial intelligence systems have demonstrated improved symptom interpretation compared with traditional rule-based symptom checker algorithms, particularly for chest pain evaluation and transient neurological symptoms.^81^ These findings suggest that language-based diagnostic models may reduce diagnostic delay by enhancing early symptom recognition and facilitating appropriate clinical referral.

Despite these promising results, multiple investigations emphasize caution against overreliance on ChatGPT for definitive diagnostic decision-making.^82,83^ Hallucination bias, the generation of plausible yet factually incorrect information, remains a significant concern. Non-deterministic output generation further undermines reproducibility, substantially limiting reliability in high-stakes diagnostic contexts.

Bias introduced through imbalanced training datasets represents another critical limitation.^84^ Studies have demonstrated significantly reduced diagnostic accuracy in underrepresented populations using AI-based dermatological lesion classification models, a concern likely applicable to language-based diagnostic systems. Furthermore, ChatGPT lacks direct access to real-time physiological monitoring data, medical imaging, and electrophysiological waveform analysis, which remain essential for definitive cardiovascular and cerebrovascular diagnosis.

### C. HIV/AIDS

Human immunodeficiency virus (HIV) infection remains a major global public health challenge, with timely diagnosis serving as the critical gateway to antiretroviral therapy, transmission prevention, and long-term disease management.^85^ Unlike many non-communicable diseases, HIV/AIDS diagnosis is fundamentally dependent on laboratory confirmation; however, clinical reasoning plays a decisive role in determining who should be tested, when testing should occur, and how results are interpreted within an appropriate clinical context.^86^ Despite the availability of highly sensitive serological and molecular assays, delayed or missed HIV diagnosis persists, particularly during acute infection and among marginalized populations, driven by nonspecific symptom presentations, pervasive stigma, low individual risk perception, and limited access to confidential testing services.^87^ Within this diagnostic landscape, large LLMs, particularly ChatGPT, have been explored as adjunctive tools to support diagnostic decision-making, testing pathway navigation, and patient engagement, rather than as substitutes for definitive laboratory diagnostics (Fig. 3).

Acute HIV infection frequently presents with nonspecific, influenza-like constitutional symptoms, including fever, maculopapular rash, generalized lymphadenopathy, pharyngitis, and myalgia, which overlap substantially with common viral illnesses and contribute significantly to underdiagnosis during the critical window of peak infectivity.^88^ Although no large-scale studies have directly evaluated ChatGPT's diagnostic accuracy specifically for acute HIV infection, several investigations of its infectious disease and internal medicine clinical reasoning provide relevant indirect evidence regarding its potential utility in this domain.^77^

Evaluation of ChatGPT's higher-order clinical reasoning across internal medicine scenarios, including infectious diseases with symptom overlap characteristic of acute HIV, demonstrated the strongest performance in syndromic pattern recognition, frequently identifying the need for HIV testing when presented with compatible symptom clusters, while appropriately refraining from asserting a definitive diagnosis without laboratory confirmation.^17^ Similarly, studies report that ChatGPT tends to generate appropriately broad differential diagnoses and emphasize the necessity of confirmatory serological or molecular testing in conditions characterized by nonspecific early manifestations, a diagnostic approach particularly appropriate for HIV evaluation.^7,89,90^

Further supporting evidence comes from examinations of ChatGPT's performance on standardized licensing-style medical questions. Assessment demonstrated that ChatGPT achieved near-passing performance on the United States Medical Licensing Examination (USMLE), including questions addressing acute retroviral syndrome recognition, diagnostic testing algorithm selection, and opportunistic infection patterns characteristic of advanced HIV disease.^18^ Collectively, these studies suggest that ChatGPT can recognize clinical presentations warranting HIV diagnostic evaluation, although its role remains appropriately limited to diagnostic suspicion prompting and testing recommendation rather than definitive diagnosis.

Risk-based testing constitutes the epidemiological cornerstone of HIV diagnosis, as contemporary testing recommendations are primarily guided by behavioral risk factors, demographic characteristics, and epidemiological exposure patterns rather than symptomatology alone.^91–95^ Multiple studies have examined ChatGPT's ability to synthesize patient-reported exposure histories and recommend appropriate screening strategies consistent with public health guidelines.^74,96^

Assessment of ChatGPT's performance in preventive medicine counseling, including sexually transmitted infection risk evaluation, found high concordance with Centers for Disease Control and Prevention (CDC) and World Health Organization (WHO) guidelines when explicit risk factors including condomless sexual exposure, men who have sex with men (MSM), injection drug use history, or prior sexually transmitted infections were clearly provided in clinical scenarios.^96^ In simulated clinical encounters, ChatGPT consistently recommended HIV screening protocols and follow-up testing strategies appropriately aligned with established diagnostic window periods and serological detection thresholds.

Studies further demonstrated that ChatGPT's responses to HIV exposure inquiries and testing questions were generally more guideline-consistent, medically accurate, and less stigmatizing than information obtained through conventional web search engines. This finding carries substantial diagnostic significance, as stigma, fear of judgment, and confidentiality concerns represent well-documented barriers to HIV testing uptake across diverse populations.^97,98^ In parallel, broader evaluations of AI-based symptom assessment platforms and conversational diagnostic agents indicate that language-based models consistently outperform traditional rule-based checker tools in accurately identifying individuals who meet established criteria for HIV testing, particularly in primary care and community-based healthcare settings.^99–101^

Beyond identifying testing indications, a comprehensive HIV/AIDS diagnosis requires understanding which specific diagnostic assays to employ, how to interpret results within a clinical context, and how to navigate complex testing algorithms, particularly in relation to immunological window periods and confirmatory testing sequences.^94^ Evidence demonstrates that ChatGPT performs well when responding to guideline-anchored clinical questions, including appropriate use of fourth-generation antigen/antibody combination assays, the role of HIV RNA nucleic acid testing in suspected early infection, and interpretation of Western blot or HIV-1/HIV-2 antibody differentiation assays.^92^ This suggests potential utility in guiding both clinicians and patients through standard diagnostic pathways and testing sequence rationale.

However, significant limitations become apparent in more diagnostically complex scenarios. Studies demonstrated that ChatGPT's accuracy substantially declines when multiple heterogeneous data elements must be longitudinally integrated, such as interpreting discordant results between screening and confirmatory tests, explaining indeterminate serological findings, or managing cases involving elite controllers or post-treatment controllers with atypical laboratory profiles.^21,102^ Given that false-negative results during the acute infection window period and discordant assay patterns remain common diagnostic challenges in HIV testing algorithms, these limitations significantly constrain ChatGPT's capacity for independent diagnostic reasoning without expert clinical oversight.

Several studies highlight ChatGPT's potential indirect diagnostic impact through enhanced patient engagement and accessible health education, both of which strongly influence HIV testing uptake and early diagnosis rates.^101^ Research found that AI-generated patient education materials addressing HIV transmission, testing procedures, and result interpretation were more rapidly produced and linguistically accessible compared to traditional expert-authored content, though occasionally demonstrated reduced technical precision. In the HIV/AIDS diagnostic context, provision of accessible, nonjudgmental, culturally appropriate explanations regarding testing rationale, window periods, confidentiality protections, and transmission risk may meaningfully encourage individuals who would otherwise avoid healthcare engagement to seek diagnostic testing services.^98^

Earlier digital health intervention studies demonstrated that conversational AI tools can reduce perceived stigma, improve honest symptom disclosure, enhance comfort discussing sexual health topics, and increase HIV testing rates among high-risk populations.^101^ ChatGPT represents a substantial technological evolution of these earlier systems, offering more sophisticated context-aware responses, natural conversational flow, and adaptive interactive guidance. Nevertheless, documented inaccuracies in nuanced diagnostic explanations, potential for generating misleading information, and absence of real-time clinical judgment remain significant concerns, underscoring the continuing need for alignment with authoritative public health information sources and clinical validation.^102^

Despite promising applications, ChatGPT exhibits critical limitations specifically relevant to HIV/AIDS diagnosis. Documentation of the hallucination phenomenon in large language models reveals that these systems may generate confident yet factually incorrect medical statements without explicit uncertainty acknowledgment.^21^ In HIV diagnostic contexts, such errors could result in false reassurance regarding infection status, unnecessary psychological distress, delayed testing, or inappropriate risk behavior modification based on inaccurate information.

Additionally, algorithmic bias in AI systems represents a substantial concern with direct implications for HIV diagnosis. Studies have demonstrated that models trained predominantly on data from high-income healthcare settings may inadequately reflect HIV epidemiology, transmission dynamics, healthcare access barriers, and diagnostic infrastructure limitations in low- and middle-income countries, where the overwhelming majority of global HIV burden exists.^103,104^ Such geographic and demographic bias could perpetuate diagnostic health disparities rather than ameliorate them.

Ethical and legal analyses further emphasize the current absence of clear regulatory frameworks and clinical accountability structures for AI-assisted diagnostic errors, reinforcing the imperative for continuous clinician oversight and the development of appropriate governance mechanisms.^105^ ChatGPT should be conceptualized and operationalized strictly as a supplementary tool for enhancing HIV testing awareness, facilitating diagnostic pathway navigation, reducing stigma-related barriers, and improving patient education, while operating under mandatory professional supervision rather than functioning as an independent diagnostic decision-making system. Only through measured, rigorously evaluated, evidence-based implementation with robust clinical oversight, continuous performance monitoring, and explicit limitation communication can LLMs meaningfully contribute to improving HIV/AIDS diagnostic outcomes while uncompromisingly maintaining patient safety, diagnostic accuracy, and clinical accountability standards.

### D. Diabetes

Diabetes mellitus is a highly prevalent metabolic disorder with a substantial proportion of cases remaining undiagnosed due to its insidious onset and prolonged asymptomatic phase.^106–108^ Accurate diagnosis relies on standardized biochemical criteria, including fasting plasma glucose, oral glucose tolerance testing, and glycated hemoglobin (HbA1c), interpreted within appropriate clinical contexts (Fig. 3).^107–110^ Unlike artificial intelligence systems designed for image analysis or physiological signal processing, ChatGPT is a language-based large language model, and its diagnostic relevance in diabetes lies primarily in its capacity to encode diagnostic knowledge, support clinical reasoning, interpret guidelines, and facilitate structured screening and triage processes rather than independently confirming disease.^7,89,90^

Several published studies have directly evaluated ChatGPT's knowledge accuracy and reasoning ability in diabetes-related contexts. Huang *et al.* assessed ChatGPT's responses to common diabetes questions and misconceptions and found that the model demonstrated moderate to high accuracy for fundamental diagnostic concepts, including definitions of diabetes and prediabetes, interpretation of HbA1c thresholds, and indications for confirmatory testing.^111^ However, endocrinology experts noted that responses occasionally lacked contextual nuance, particularly in scenarios where diagnostic interpretation depends on comorbid conditions such as anemia, chronic kidney disease, or recent acute illness.^111^ Similar findings were reported in a Turing test–inspired survey conducted among healthcare professionals, where ChatGPT-generated answers to diabetes-related questions were frequently indistinguishable from those written by human experts, suggesting strong baseline diagnostic knowledge but not necessarily superior clinical judgment.^112^

Formal examination-based evaluations further support these observations. In assessments using multiple-choice questions in endocrinology and diabetes technology, ChatGPT achieved correct response rates of approximately 55%–60%, performing adequately on core diagnostic criteria while struggling with questions requiring higher-order reasoning or subtype differentiation.^18,113^ Studies evaluating ChatGPT's performance on licensing-style medical examinations similarly showed that the model reliably recalled diagnostic thresholds and screening indications but exhibited reduced accuracy when clinical ambiguity was introduced.^114^ These findings indicate that ChatGPT performs best when diagnostic rules are explicit and standardized, as in diabetes, but less reliably when interpretation requires synthesis of longitudinal or conflicting data.

ChatGPT appears to have particular utility in diabetes screening and risk identification rather than definitive diagnosis. Several analyses have shown that the model can correctly identify individuals who meet guideline-based screening criteria by integrating age, body mass index, family history, cardiovascular risk factors, and gestational history.^115,116^ In comparative studies of AI-generated health advice, ChatGPT consistently framed diabetes testing as a preventive diagnostic measure rather than a symptom-driven response, aligning with contemporary screening recommendations.^115^ This capability may be especially relevant in primary care and public-facing digital health environments, where under-screening remains a major contributor to delayed diagnosis.

The model has also been evaluated for its ability to interpret laboratory results related to diabetes diagnosis. Kusunose *et al.* demonstrated that ChatGPT could accurately explain isolated fasting glucose or HbA1c values according to established thresholds.^117^ However, Mahuli *et al.* reported diminished reliability when multiple laboratory values required reconciliation, such as discordant HbA1c and glucose measurements or fluctuating results over time.^118^ These limitations underscore that while ChatGPT can contextualize individual test results, it lacks the capacity to resolve diagnostic uncertainty that depends on temporal trends, repeated testing, or integration with physical examination findings.

Diagnostic performance is further limited when considering diabetes heterogeneity. Across multiple studies, ChatGPT showed difficulty distinguishing between diabetes subtypes, particularly latent autoimmune diabetes in adults (LADA), monogenic diabetes, and secondary forms related to endocrinopathies or medication exposure.^119,120^ While the model accurately described textbook distinctions between type 1 and type 2 diabetes, it frequently defaulted to generic classifications when confronted with atypical presentations, raising concerns about misclassification at diagnosis and subsequent therapeutic implications.^120^

Beyond technical reasoning, several studies highlight the potential indirect diagnostic impact of ChatGPT through improved patient engagement and education. Research evaluating AI-generated educational content found that ChatGPT-produced explanations of diabetes testing and disease mechanisms were generally understandable and accessible, particularly for individuals with lower health literacy.^74,121^ Although these studies did not measure diagnostic accuracy directly, they suggest that conversational AI may increase screening uptake and early diagnostic engagement by clarifying when and why testing is necessary. Earlier digital triage research supports this hypothesis, demonstrating that conversational interfaces encourage disclosure of symptoms and risk factors relevant to metabolic disease detection.^99,122^

### E. Rare and complex disease

Rare diseases (RDs) collectively affect over 400 × 10^6^ individuals worldwide, encompassing more than 7000 distinct disorders, approximately 80% of which have genetic origins.^123–125^ Despite their collective prevalence, RDs present formidable diagnostic challenges characterized by prolonged diagnostic odysseys averaging 5 years in Europe and 6–7 years in the United States, attributable to disease rarity, phenotypic heterogeneity, and limited clinical familiarity.^126–128^ Fewer than 5% of RDs have FDA-approved therapies, leaving more than 6500 conditions without targeted interventions.^129^ Tragically, 30% of affected children do not survive beyond age 5.^130^ Within this diagnostically challenging landscape, large language models (LLMs), particularly ChatGPT, have emerged as potential adjunctive tools to support clinical reasoning, facilitate information synthesis, and accelerate diagnostic pathways.^7,131,132^

Rare diseases frequently manifest with nonspecific, overlapping, or atypical symptom presentations that challenge timely recognition, particularly in primary care settings.^127^ ChatGPT's natural language processing (NLP) capabilities enable the interpretation of unstructured, patient-reported symptom narratives and systematic differential diagnosis generation.^132^ Multiple NLP-based approaches have demonstrated utility in extracting clinical phenotypes from unstructured electronic health record (EHR) data.^133,134^ Deep learning models, including BioBERT and ClinicalBERT, achieved F1-scores of 0.85 for extracting rare disease signs and symptoms from clinical text.^135^

Comparative evaluations showed ChatGPT achieved F1-scores of 0.472 in zero-shot phenotype extraction and 0.610 with few-shot learning when processing clinical cases from the RareDis corpus, though specialized models like BioClinicalBERT demonstrated superior performance (F1-score 0.689).^136^ This performance differential underscores that while ChatGPT demonstrates baseline competency, domain-specific fine-tuned models currently outperform general-purpose LLMs for structured phenotype extraction.^136^

Nevertheless, ChatGPT's conversational interface and ability to process free-text symptom descriptions may provide value in patient-facing applications and preliminary clinical assessment where structured phenotype ontologies are unavailable.78 Evaluation using medical licensing examination questions addressing rare genetic disorders reported near-passing performance, suggesting baseline competency in recognizing patterns warranting specialized investigation.^137^

Accurate rare disease diagnosis increasingly depends on systematic integration of clinical phenotypes with genomic variants and disease-specific knowledge resources.^138,139^ RareDxGPT, an enhanced ChatGPT model integrating external knowledge from the RareDis Corpus covering 717 rare diseases, achieved diagnostic accuracy of 40%–43% across various prompting strategies when evaluated on 30 clinical cases. While suboptimal for autonomous diagnosis, this demonstrates proof-of-concept for augmenting LLMs with disease-specific knowledge bases.^136^

More sophisticated approaches combining LLMs with structured knowledge graphs have shown greater promise. SHEPHERD, a few-shot learning model integrating deep learning with knowledge graphs, ranked the correct causal gene first in 40% of Undiagnosed Diseases Network (UDN) cases, with 77.8% ranked in the top five for patients with atypical presentations.^140^ PhenoBrain, an AI pipeline extracting phenotypes from EHRs, demonstrated performance outperforming specialist physicians in diagnostic ranking when evaluated on cohorts containing 431 rare diseases.^141^

These specialized systems illustrate that optimal rare disease diagnostic support requires integration with curated phenotype ontologies (Human Phenotype Ontology), variant databases (ClinVar, gnomAD), and disease classifications (Orphanet, OMIM).^142^ ChatGPT's role is most appropriately conceptualized as facilitating phenotype documentation and hypothesis generation rather than performing definitive molecular diagnosis.^132^

A case study documented ChatGPT's application in facilitating the diagnosis of Alström syndrome (prevalence 1–9 per 1 000 000) in a patient with a complex medical history. AI-assisted text analysis helped identify ALMS1 as the candidate gene, enabling diagnostic confirmation within two business days.^143^ While representing a single case with inherent limitations, this demonstrates how LLMs can accelerate literature review and gene prioritization under expert supervision.^143^

Broader evaluation using the RAMEDIS dataset showed DxGPT (GPT-4 fine-tuned on medical knowledge) achieved diagnostic accuracy of 50% when analyzing patient symptoms, performing comparably to clinicians.^144^ MetaGP, a generative foundation model trained on over 8 × 10^6^ resources, achieved diagnostic scores of 1.57 when evaluated on 97 rare systemic disease cases, compared with junior practitioners (0.94), mid-level practitioners (1.02), and senior practitioners (1.5), suggesting sufficiently sophisticated models can approach senior clinician-level performance for certain tasks.^145^

However, performance exhibits substantial variability depending on task complexity and disease prevalence within training data.^21^ ChatGPT demonstrates the strongest performance for symptom-based reasoning and initial differential diagnosis generation, while accuracy diminishes for multi-step diagnostic synthesis requiring longitudinal integration of laboratory or imaging data.^21^ Evaluation using annotated clinical notes achieved an AUC of 0.960 and an F1-score of 0.927 when identifying rare disease signals, suggesting high sensitivity for flagging cases requiring further evaluation.^146^

NLP algorithms applied to EHRs have demonstrated the capacity to extract red flag symptoms, identifying patients warranting clinical review and genetic testing.^146^ Studies in hereditary transthyretin amyloidosis polyneuropathy achieved an F1-score of 0.930 when identifying high-risk patients from unstructured medical records, resulting in a 48.6% increase in genetic testing recommendations.^147^

Similar approaches have been applied to metabolic rare diseases, including Pompe disease and Fabry disease.^148^ For Fabry disease screening, spaCy-based NLP pipelines achieved AUC of 0.998 when analyzing multi-hospital EHR data.^149^ These metrics suggest that when applied to well-defined screening tasks, NLP-based approaches can substantially improve case identification efficiency.^149^

ChatGPT's role in this screening context would involve processing patient-reported symptoms, organizing clinical histories, and generating preliminary assessments suggesting appropriate specialist referral.^132^ While not directly evaluated in prospective rare disease screening studies, its underlying NLP capabilities suggest potential utility as a patient-facing triage tool or clinician decision-support interface when integrated with validated screening algorithms.^78^

ChatGPT's application to rare disease diagnosis faces several fundamental constraints. Data scarcity represents the most critical limitation: rare diseases have limited case series, sparse literature, and underrepresentation in training corpora. Records, registries, and omics databases remain disconnected across institutions, hindering reproducibility and limiting evidence for model training. Training data imbalances introduce systematic biases with direct implications for diagnostic equity.^150^ Studies evaluating facial recognition AI for genetic syndrome diagnosis documented dramatic performance disparities: 100% accuracy for Down syndrome, 66.7% for Noonan syndrome, but 0% for Morquio syndrome, and 8.3% for Neurofibromatosis type 1 in an admixed Colombian population, compared to substantially higher performance in European ancestry populations. Such biases could perpetuate rather than ameliorate diagnostic health disparities. The propensity for hallucinated information, confident yet factually incorrect statements, represents a particularly acute risk in rare disease contexts where clinical evidence is sparse and erroneous information could precipitate inappropriate testing or delayed intervention. Privacy risks are amplified in rare disease populations where small cohort sizes enable potential re-identification despite anonymization.^151^

Furthermore, ChatGPT lacks direct integration with essential diagnostic infrastructure, including genetic databases, variant interpretation algorithms, phenotype-matching systems, and medical imaging archives.^132^ The model cannot independently analyze sequencing data, assess variant pathogenicity, or access patient-specific longitudinal clinical data. The rapidly evolving landscape of gene-disease associations and variant reclassifications necessitates continuous knowledge updating exceeding the capabilities of models with fixed training cutoff dates. ChatGPT should be conceptualized strictly as a supplementary cognitive aid under continuous expert oversight rather than an autonomous diagnostic system.^21^ Appropriate implementation requires prospective validation across diverse populations, robust governance frameworks delineating use boundaries, transparent communication of limitations, and continuous performance monitoring.

Optimal integration will likely involve hybrid architectures combining LLMs' natural language understanding with specialized rare disease resources.^7,152,153^ Emerging approaches include federated learning, enabling privacy-preserving collaborative training across distributed cohorts (improving predictive accuracy by 13.3% compared to local models), transfer learning, adapting models pretrained on common diseases to rare disease applications, and explainable AI, providing transparent reasoning pathways.^154–156^

AutoMAxO, leveraging LLMs to automate curation of treatments for rare diseases, extracted 18 631 candidate annotations with 538 confirmed across 21 rare diseases, demonstrating how LLMs can accelerate knowledge curation. Knowledge graph-based systems like mediKanren illustrate potential for data-driven precision care when LLM capabilities combine with structured biomedical knowledge representation.^157,158^ Future priorities include establishing FAIR (Findable, Accessible, Interoperable, Reusable) data infrastructures, implementing standardized phenotype ontologies enabling cross-institutional model training, developing adaptive trial designs appropriate for small populations, and embedding equity as a core design principle to ensure reliable performance across diverse ancestry groups, geographic regions, and healthcare settings.^138,159–161^

## III. ROLE OF ChatGPT IN DECISION-MAKING IN PRECISION MEDICINE

Key roles of ChatGPT in clinical decision-making for precision medicine are shown in Table II. Clinical decision support (CDS) systems utilize technology and data analytics to provide healthcare professionals with contextually relevant information and evidence-based recommendations.^162,163^ Traditional CDS architectures rely on rule-based algorithms and predefined decision trees, which demonstrate limited flexibility when confronted with the phenotypic heterogeneity and molecular complexity characteristic of precision medicine.^162,163^ ChatGPT's transformer-based architecture enables natural language understanding and generation capabilities that facilitate interactive, context-aware dialogue supporting exploration of complex clinical scenarios.^164^

**TABLE II.** Role of ChatGPT in clinical decision-making for precision medicine.

Domain	Role of ChatGPT	Key Evidence/Examples	Strengths	Limitations
Clinical decision support (general)	Conversational, context-aware augmentation of traditional CDS systems	LLMs outperform rigid rule-based CDS in flexible reasoning and synthesis	Interactive dialogue; rapid knowledge synthesis	Lack of causal inference; output variability
Genomic variant interpretation	Synthesis of structured genomic findings and variant frameworks	ChatGPT-assisted ALMS1 prioritization enabled Alström syndrome diagnosis	Accelerates rare disease diagnosis	Cannot analyze raw NGS data
Precision treatment planning	Mapping molecular alterations to evidence-based therapies	Targeted therapy matching and trial identification in oncology	Supports biomarker-driven therapy	Limited individualized risk–benefit modeling
Knowledge graph–integrated AI systems	Linking genes, drugs, and diseases for therapy proposals	mediKanren; AutoMAxO (538 validated annotations)	Scalable hypothesis generation	Requires expert validation
Patient education and communication	Translation of genomic data into patient-friendly language	Improved readability vs expert-written materials	Enhance health literacy	Reduced technical precision

Precision medicine diagnosis increasingly depends on accurate interpretation of genomic variants identified through next-generation sequencing.^165^ While ChatGPT cannot directly analyze raw sequencing data, the model can assist in synthesizing variant interpretation frameworks when provided with structured genomic information.^166^ Specifically, ChatGPT can help navigate classification criteria, retrieve functional annotations from databases, identify genotype-phenotype correlations from literature, and flag potentially actionable variants. A documented case study described ChatGPT-assisted diagnosis of Alström syndrome, where AI-driven text analysis of complex medical history helped prioritize ALMS1 as a candidate gene, enabling rapid diagnostic confirmation.^167^

Precision medicine treatment planning requires integration of molecular diagnostic information with pharmacogenomic data, drug interactions, patient-specific contraindications, and evidence regarding therapeutic efficacy in molecularly defined patient subsets.^168^ ChatGPT can support this by mapping identified molecular alterations to evidence-based therapeutic options, considering patient demographics, comorbidity burden, prior treatments, and tumor mutational profiles.^168^ For oncology applications, ChatGPT can assist in identifying targeted therapies matched to actionable mutations, exploring combination strategies, assessing clinical trial eligibility, and retrieving information regarding resistance mechanisms.^169^

Knowledge graph-based systems integrating LLM capabilities, such as mediKanren, have demonstrated utility in linking diseases, drugs, and genes to propose personalized therapeutic options.^170,171^ Platforms like CURATE.AI have shown clinical validation for AI-guided personalized dosing optimization, dynamically adjusting targeted therapy based on real-time patient data to maximize efficacy while minimizing toxicity.^172^ AutoMAxO, leveraging LLMs with structured prompts, demonstrated the capacity to automate treatment curation for rare diseases, extracting 18 631 candidate annotations with 538 confirmed across 21 conditions.^171^

The rapidly evolving precision medicine evidence base encompassing updated treatment guidelines, emerging biomarker discoveries, newly approved targeted agents, and accumulating real-world evidence creates information currency challenges.^168^ ChatGPT can provide real-time access to current clinical practice guidelines, synthesize recommendations across potentially conflicting sources, and contextualize guideline applicability to individual patient scenarios.^173^ For example, when evaluating treatment options for advanced non-small cell lung cancer harboring specific driver mutations, ChatGPT can retrieve guideline recommendations, summarize FDA-approved targeted therapies with biomarker requirements, identify clinical trial options, and highlight emerging therapeutic strategies from recent literature.^169^

Precision medicine requires effective communication of complex genetic and molecular information to patients and facilitation of informed consent for genomic testing.^164^ ChatGPT's conversational capabilities enable the provision of personalized patient education materials, responses to questions regarding genetic test results or treatment options, and translation of technical terminology into accessible language appropriate to individual health literacy levels.^164^ Studies comparing AI-generated patient education materials with expert-authored content found that ChatGPT produced information rapidly with enhanced accessibility, though sometimes with reduced technical precision. Such patient-facing applications could enhance health literacy, empower informed participation, and improve satisfaction with precision medicine care delivery.^166^

**FIG. 4.**
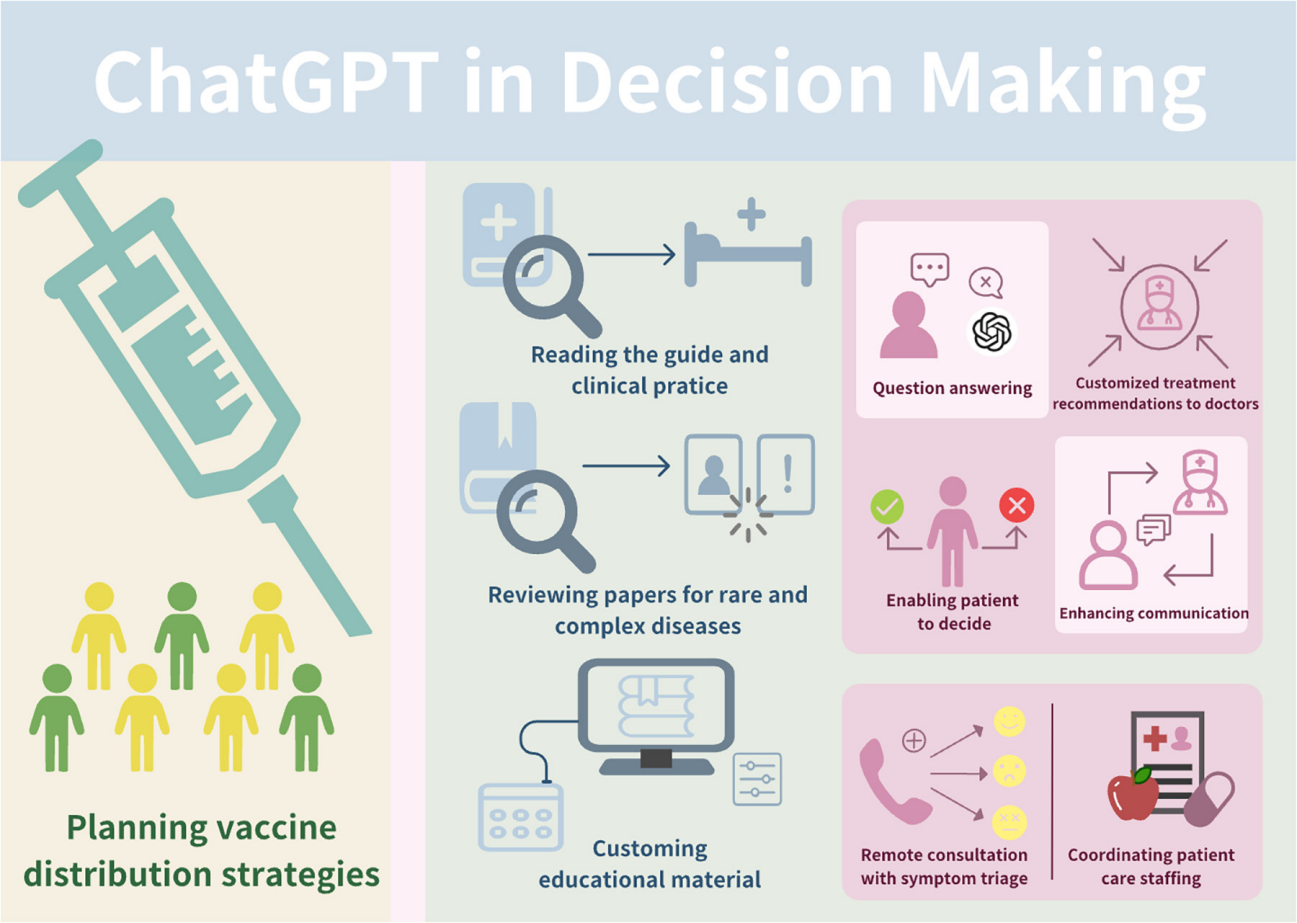
ChatGPT in decision making.

Recent empirical studies have systematically evaluated ChatGPT's performance in clinical decision-making across diverse medical specialties, revealing both promising capabilities and critical limitations.^173^ In otorhinolaryngology, prospective evaluation of 20 reality-inspired clinical cases demonstrated that ChatGPT achieved a mean score of 4.4 out of 5 (SD 1.2), significantly lower than the otorhinolaryngologist consensus score of 4.91 (SD 0.3; p < 0.001), with notable temporal instability evidenced by discordant responses in 25% of repeated queries. Similarly, assessment of ChatGPT's performance against multidisciplinary Heart Team decisions for coronary revascularization in 128 patients revealed suboptimal concordance, with ChatGPT 01 demonstrating 82% sensitivity for identifying coronary artery bypass grafting candidates but only 43.7% sensitivity for percutaneous coronary intervention, and Cohen's kappa values of 0.17 and 0.03 for the two ChatGPT versions, indicating poor overall agreement with expert consensus. More favorable results emerged in specific clinical contexts: ChatGPT achieved 77% agreement with Heart Team decisions regarding severe aortic stenosis management across 150 patients, with agreement rates of 90% for transcatheter valve implantation but only 65% for surgical valve replacement and medical treatment; in head and neck oncology, comparative analysis against National Comprehensive Cancer Network (NCCN) guidelines across 68 hypothetical cases and 204 clinical scenarios demonstrated high sensitivity and overall accuracy for primary treatment, adjuvant therapy, and follow-up recommendations, though with notable inaccuracies in certain primary treatment scenarios. Evaluation in renal cell carcinoma multidisciplinary tumor board decision-making showed 62.1% agreement with expert decisions (*κ* = 0.44, p < 0.001), with the highest concordance when follow-up imaging was recommended and significant variation based on disease status.^174^ Performance assessment using the Iranian medical licensing examination revealed that ChatGPT correctly answered 68.5% of 200 questions, substantially exceeding the 45% passing threshold and significantly outperforming random guessing with superior performance in decision-making categories compared to basic science evaluation and diagnostics, though remaining inferior to human physician performance.^174^ Collectively, these studies demonstrate that ChatGPT exhibits variable performance across clinical specialties and decision contexts, with capabilities ranging from near-expert concordance in guideline-aligned scenarios to substantial discordance in complex, multifactorial treatment decisions requiring integration of imaging data, nuanced clinical context, and individualized risk-benefit assessment, underscoring the necessity for continuous expert oversight and validation before clinical deployment (Fig. 4).

## IV. STATE-OF-THE-ART ChatGPT TECHNOLOGIES FOR PERSONALIZED MEDICINE

ChatGPT, based on the generative pre-trained transformer (GPT) architecture, marks a major advance in large language models (LLMs) for personalized medicine. Trained through unsupervised learning on large text corpora and refined via reinforcement learning from human feedback (RLHF), it enables context-aware and human-like text generation without task-specific tuning.^163,175,176^ In personalized medicine, ChatGPT supports clinical decision-making, patient communication, pharmacogenomic interpretation, and care coordination. Nonetheless, variable performance across medical domains underscores the need for rigorous clinical validation and assessment of its technical and practical limitations.^177^

**FIG. 5.**
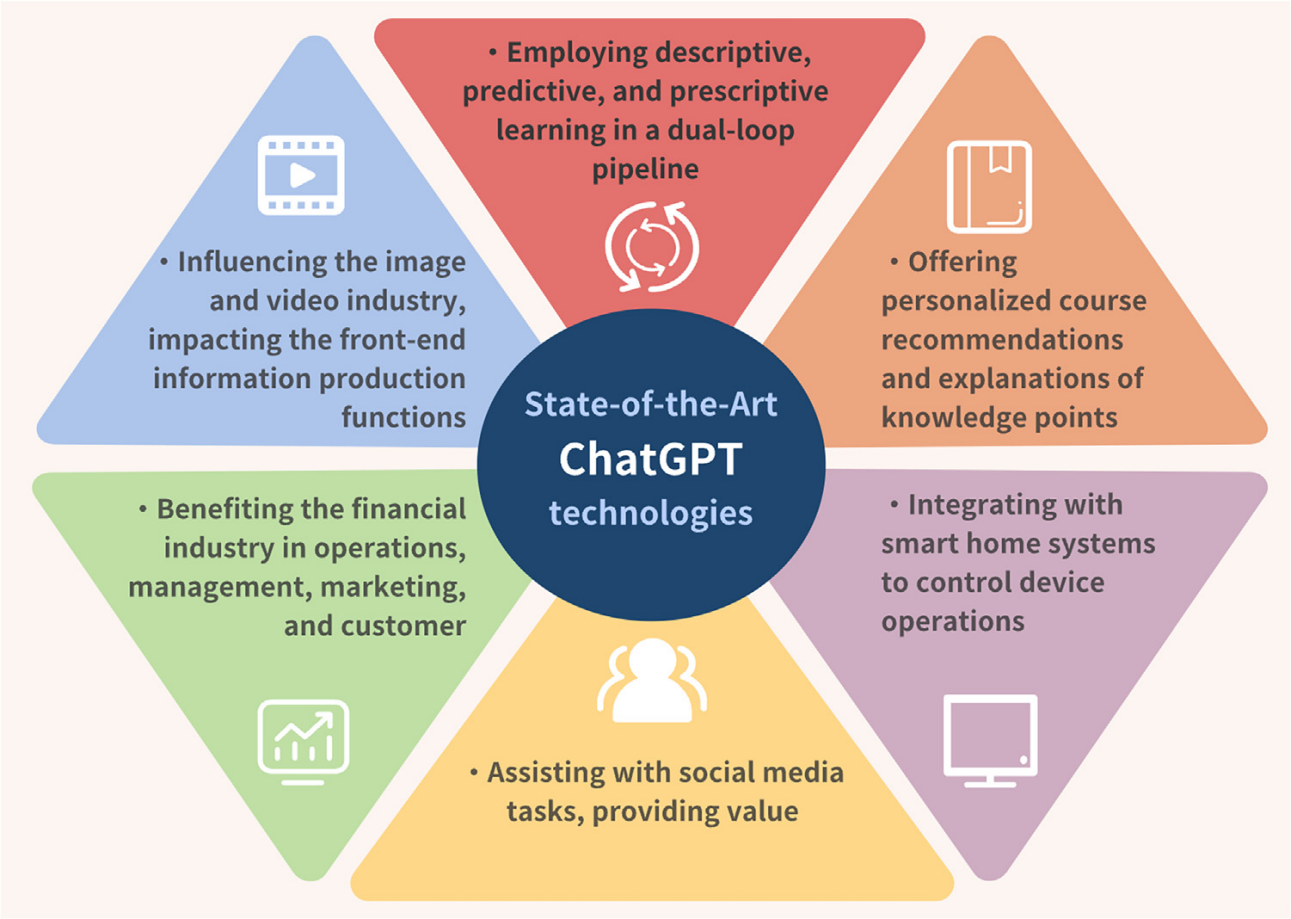
State-of-the-art technologies of ChatGPT.

ChatGPT's architecture is built on transformer decoder blocks employing self-attention mechanisms, where relationships between input tokens are computed using query-key-value (QKV) operations.^177^ This design captures long-range dependencies in clinical narratives, genomic data, and complex medical reasoning tasks (Fig. 5). GPT-4, the most advanced public version, features extended context windows (>128 K tokens in GPT-4 Turbo), enabling single-pass analysis of comprehensive electronic health records, longitudinal patient histories, and large clinical documents.^178^

Key innovations for personalized medicine include in-context learning (ICL), allowing zero- or few-shot task adaptation through structured prompting, and chain-of-thought (CoT) prompting, which supports stepwise clinical reasoning. Multimodal extensions such as GPT-4V incorporate visual inputs from radiology, pathology, or wearable data alongside text, though performance remains variable for domain-specific imaging.^178^

ChatGPT processes clinical text via tokenization (byte-pair encoding) and contextual embedding generation across transformer layers with residual connections and layer normalization for training stability. These features enable the extraction of medical entities and the generation of clinically meaningful responses, although limitations persist regarding factual consistency, temporal reasoning, and hallucination control.^179,180^

### A. Applications in clinical decision support

Empirical evaluations of ChatGPT in clinical decision support reveal considerable variability in accuracy and concordance across specialties. In otorhinolaryngology, analysis of 20 realistic clinical cases showed ChatGPT achieved a mean score of 4.4 ± 1.2, significantly lower than expert consensus (4.91 ± 0.3; p < 0.001), with 25% response discordance upon repeated queries, indicating temporal instability and reproducibility concerns.^181^

Cardiovascular assessments demonstrate performance dependent on case complexity. In 128 coronary revascularization cases, ChatGPT-o1 achieved 82% sensitivity for identifying coronary artery bypass grafting candidates but only 43.7% for percutaneous coronary intervention, with poor agreement with Heart Team decisions (*κ*  =  0.17 for o1; *κ*  =  0.03 for 4o). In severe aortic stenosis, ChatGPT showed 77% overall agreement across 150 patients, with 90% concordance for transcatheter valve implantation but only 65% for surgical replacement and conservative management.^182^

Oncological evaluations showed moderate but clinically relevant performance. When benchmarked against NCCN guidelines for head and neck cancers across 68 cases and 204 scenarios, ChatGPT demonstrated high sensitivity for primary, adjuvant, and follow-up therapy recommendations, though inconsistencies persisted in certain primary treatment contexts. In renal cell carcinoma tumor board simulations, ChatGPT matched expert consensus in 62.1% of cases (*κ*  =  0.44, p < 0.001), performing best in follow-up imaging recommendations but variably across disease stages and treatment complexity.^183^

Performance on standardized examinations offers further benchmarking. On the Iranian medical licensing exam, ChatGPT answered 68.5% of 200 questions correctly, exceeding the 45% pass mark and outperforming random guessing while performing better in decision-making than in diagnostic or basic science domains. Despite encouraging results, its clinical reasoning and diagnostic precision remain inferior to those of physicians.

### B. Patient engagement and education

ChatGPT shows significant potential in patient education and engagement. Studies demonstrate that it rapidly generates accessible, readable materials compared to traditional resources, although occasional reductions in technical precision occur relative to expert-authored content. Its conversational interface enables personalized explanations suited to individual health literacy, cultural context, and patient-specific needs.^184^

In medication adherence support, ChatGPT can explain treatment rationales, side effects, timelines, and adverse event management through natural dialogue. Integration into medication management systems has improved patient compliance and satisfaction, though direct causal attribution to ChatGPT requires prospective validation. The model can also provide individualized lifestyle recommendations based on comorbidities, diet, culture, and activity level, supporting behavioral modification.^185^

However, patient-facing applications raise concerns regarding accuracy, health literacy, and safe use. While enhancing understanding and empowerment, ChatGPT may generate inaccurate or misleading information, risking inappropriate reassurance or delayed care. Responsible deployment requires clear disclaimers, defined advisory limits, and explicit prompts for professional medical consultation.

### C. Pharmacogenomic interpretation and precision therapeutics

Pharmacogenomics exemplifies the intersection of ChatGPT's language understanding with precision therapeutics. When provided with structured genotype data (CYP2C19, CYP2D6, TPMT, and SLCO1B1), ChatGPT can reference Clinical Pharmacogenetics Implementation Consortium (CPIC) guidelines, identify clinically validated gene–drug pairs, and explain results in patient-accessible language.^176^

Studies comparing ChatGPT outputs with CPIC recommendations report ∼89% concordance, indicating baseline competency for well-established gene–drug associations. Performance declines, however, for novel variants, multigene interactions, and cases requiring integration of clinical factors such as comorbidities, concurrent medications, or organ function.^177^

Key limitations include (1) inability to process raw genomic or VCF data without preprocessing; (2) dependence on outdated training data, limiting awareness of recent evidence; (3) lack of real-time linkage to drug interaction or clinical databases; and (4) potential generation of plausible but inaccurate interpretations, especially for rare variants or understudied populations.

### D. Remote monitoring and electronic health record integration

Integration of ChatGPT with remote patient monitoring and electronic health record (EHR) systems presents major opportunities and technical challenges. Potential applications include summarizing longitudinal patient data, extracting clinically relevant information from unstructured notes, detecting adverse events or deterioration trends, and automating care coordination messages.^179,180^

ChatGPT's NLP capabilities enable the extraction of medical entities, symptoms, diagnoses, medications, labs, and clinical events—from free-text documentation. Transformer-based models in clinical NLP tasks (e.g., named entity recognition, relation extraction, phenotype identification) have achieved F1-scores of 0.85–0.97 depending on the dataset and entity type. However, direct evaluations of ChatGPT in real-world EHR contexts remain scarce, with most research focused on biomedical models such as BioBERT, ClinicalBERT, or domain-adapted variants.^184,186^

Integration barriers include: (1) stringent privacy and security requirements under HIPAA and GDPR; (2) absence of native interoperability with clinical systems; (3) necessity for structured formatting and text preprocessing; (4) high computational cost and latency for large datasets; and (5) critical validation needs to ensure data accuracy, completeness, and patient safety.

## V. ChatGPT IN DRUG DISCOVERY AND PERSONALIZED TREATMENT PLANNING

### A. ChatGPT and GPT models in drug discovery

**FIG. 6.**
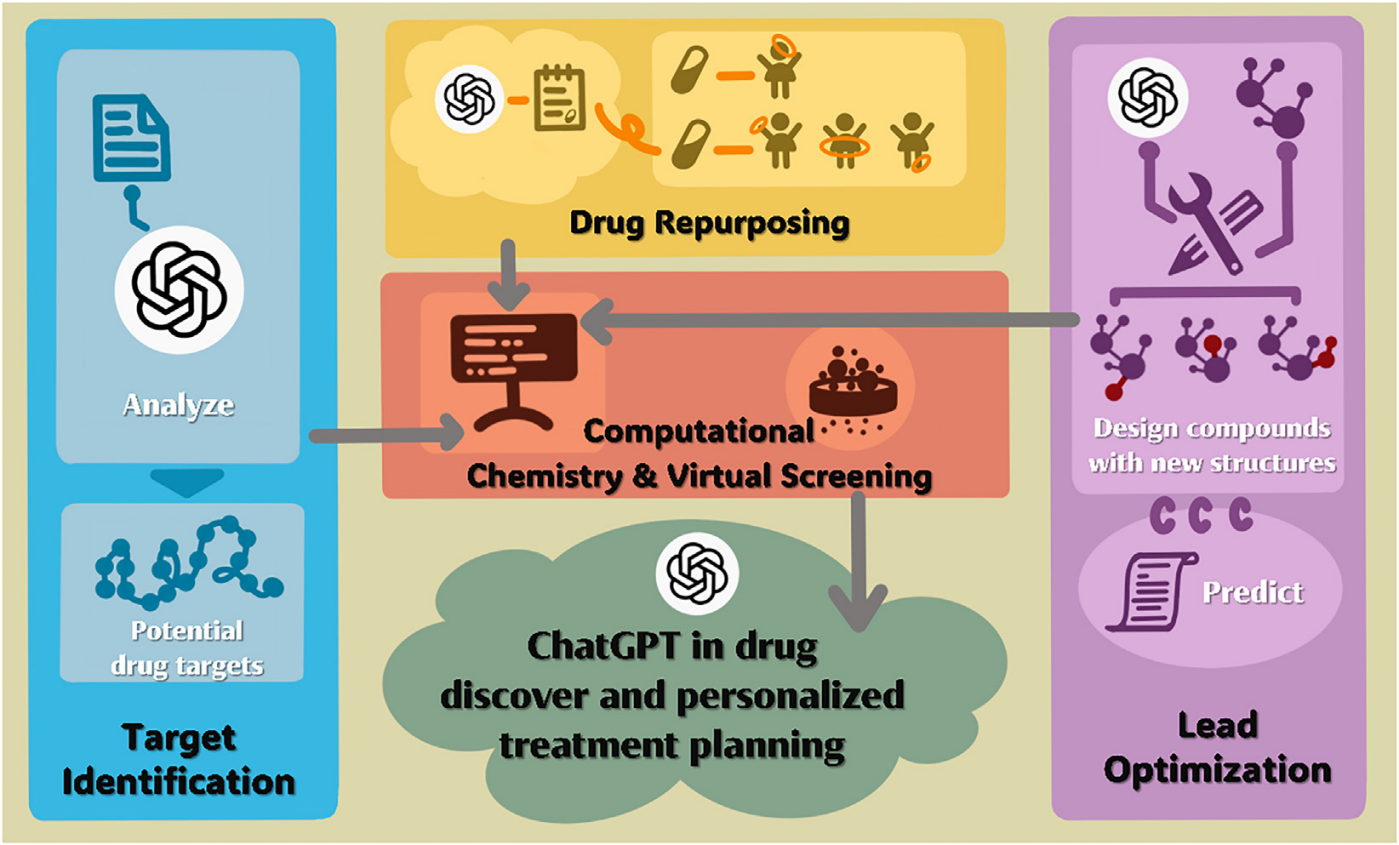
Applications of ChatGPT in drug discovery and personalized treatment planning.

Despite significant progress in biomedical research, fewer than 500 human druggable targets had been identified by 2022.^187^ Conventional drug discovery pipelines remain costly, slow, and prone to failure, driving the adoption of AI-driven strategies.^188,189^ Artificial intelligence can reduce development time and cost by 25%–50% during discovery and preclinical stages.^190^ Among AI approaches, LLMs, particularly GPT-based architectures such as ChatGPT, have emerged as powerful tools for accelerating drug discovery and development (DDD) while supporting personalized therapeutic decision-making (Fig. 6).^191^

A major limitation in AI-enabled DDD is restricted access to labeled molecular and clinical datasets due to proprietary and privacy concerns.^192^ Privacy-preserving strategies, including federated learning, differential privacy, and secure multi-party computation (MPC), enable collaborative model training across institutions without direct data sharing.^193^ In parallel, fine-tuning pre-trained GPT models on domain-specific biomedical and chemical datasets allows pharmaceutical applications to leverage foundational knowledge while minimizing computational cost and improving task-specific performance.^194^

ChatGPT and related GPT models can exploit vast unlabeled biological resources, including more than 250 × 10^6^ protein sequences in the UniProt database, to autonomously learn molecular and functional relationships.^195^ By integrating biomedical literature, patents, and clinical databases, ChatGPT facilitates rapid identification and validation of disease-associated targets, genes, and compounds. In virtual screening and early optimization, GPT-based models assist in prioritizing compounds with favorable binding affinity and optimized ADMET properties, supporting rational and patient-relevant drug design.^194^

A representative application was reported by Othman *et al.*, who developed a GPT-4-based pipeline for automated biomedical literature screening during public health emergencies. The system achieved an accuracy of 92.87% for SARS-CoV-2 and 87.40% for Nipah virus, substantially reducing manual review time and accelerating target identification, thereby enabling faster therapeutic responses during outbreaks.^196^

### B. GPT-based de novo and multi-target drug design for personalized therapy

Traditional de Novo drug design has largely focused on single molecular targets, limiting effectiveness against complex, multifactorial diseases. MTMol-GPT addresses this limitation by generating multi-target molecules using transformer networks pretrained on ChEMBL data and a generative adversarial imitation learning (GAIL) framework.^197^ The model successfully generated novel compounds targeting DRD2, EGFR, and c-Src receptors. Molecular docking and pharmacophore analyses confirmed their drug-likeness and therapeutic potential, particularly for neuropsychiatric disorders and breast cancer, highlighting relevance to personalized therapeutic strategies.^198^

SynerGPT extends GPT modeling to drug combination discovery by learning drug synergy patterns without relying on textual corpora.^199^ Using in-context learning for drug synergy (ICL-DS), SynerGPT dynamically adapts from small patient- or cancer-specific datasets (10–20 examples), enabling personalized synergy prediction. The model effectively retrieves synergistic and structurally related drugs, enhancing explainability and supporting individualized combination therapy selection.^199^

### C. Chemical space exploration and target-specific molecular design

Several GPT-based frameworks have been developed to explore previously inaccessible regions of chemical space. cMolGPT enables target-specific de novo molecular generation, producing diverse, drug-like virtual libraries aligned with real chemical distributions.^200^ ChemGPT, trained on up to 10 × 10^6^ PubChem molecules, demonstrated that neural scaling laws observed in natural language processing also apply to molecular modeling, with increasing model size improving performance and representation quality.^201,202^

DrugGPT integrates protein–ligand interaction data by jointly tokenizing protein sequences and ligands using byte pair encoding. This strategy improves training efficiency and enables ligand generation directly from protein sequences or minimal prompts. DrugGPT successfully regenerated known protein–ligand pairs and generated novel ligands for ENPP2, demonstrating its potential for innovative and target-specific ligand design relevant to personalized drug development.^200,203^

### D. GPT models in clinical trials and pharmacovigilance

Beyond discovery, GPT models are increasingly applied to clinical research and drug safety monitoring. TWIN-GPT generates personalized digital twins for virtual clinical trials, reducing cost, duration, and privacy risks. Trained on data from a Phase III breast cancer trial (NCT00174655), TWIN-GPT demonstrated high fidelity and privacy preservation, achieving a nearest neighbor adversarial accuracy (NNAA) score of 0.271.^204^

In pharmacovigilance, GPT-3.5 and GPT-4 have been used to automate literature screening for adverse drug reactions, achieving sensitivities and reproducibility exceeding 93%–97%. These applications significantly enhance post-marketing drug safety surveillance and support personalized risk assessment in real-world patient populations.^205^

## VI. ChatGPT AND MULTI-OMICS DATA INTEGRATION IN PRECISION MEDICINE

**FIG. 7.**
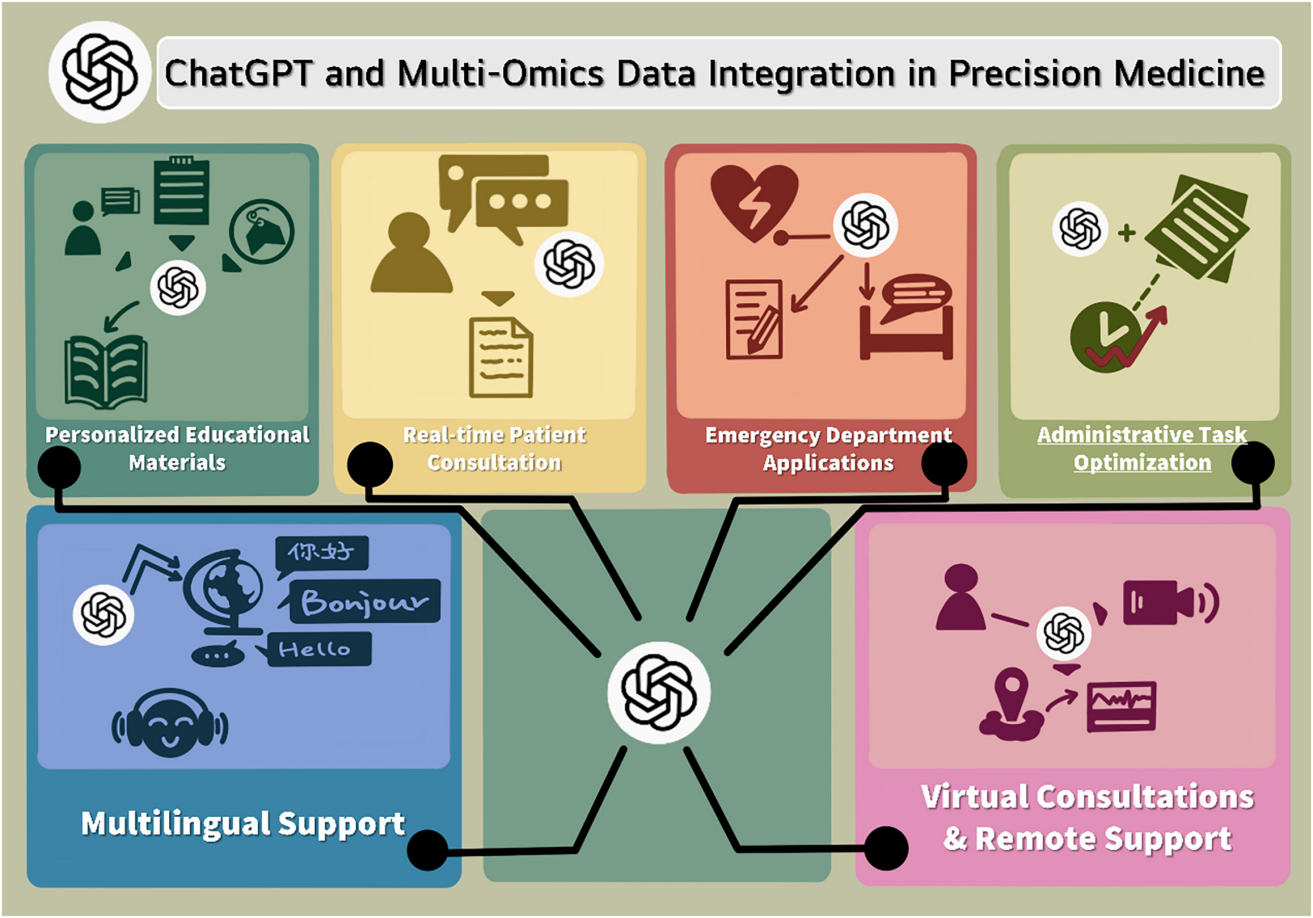
The role of ChatGPT and multi-omics data integration in precision medicine.

The integration of multi-omics data with advanced AI technologies like ChatGPT is revolutionizing precision medicine by offering a more comprehensive approach to personalized treatment. This integration combines genomics, transcriptomics, and proteomics, enabling more accurate diagnosis, prognosis, and treatment strategies.^206^ ChatGPT's potential in this field is complex, and it can interpret multifarious data sets, process scientific literature, generate novel hypotheses and analyze patient-specific data from electronic health records^207^ (Fig. 7).

Recent advancements in AI models, such as attention mechanism models and graph convolutional networks, have emerged as powerful tools in precision medicine, enhancing our ability to extract meaningful insights from multi-omics data sets.^208,209^ Notable projects like The Cancer Genome Atlas (TCGA), Human Cell Atlas (HCA), and integrated Personalized Omics Profiling (IPOP) have demonstrated the power of multi-omics data integration in advancing our understanding of human biology and disease mechanisms.^210,211^

For instance, in respiratory syncytial virus bronchiolitis research, the integration of clinical, viral, airway microbiome, transcriptome, and metabolome data identified biologically distinct endotypes with differential risks of childhood asthma development.^209^ Similarly, AI-enhanced multi-omics approaches in neurodegenerative diseases have uncovered key regulatory pathways in Alzheimer's and Parkinson's, although translating these insights into clinical applications remains complex. The synergy between ChatGPT and multi-omics data represents a significant advancement in precision medicine, holding the potential to revolutionize our understanding of disease mechanisms and the development of truly personalized treatment strategies.

## VII. CHATGPT IN PATIENT EDUCATION AND PERSONALIZED HEALTH COMMUNICATION

Patient education and effective health communication constitute fundamental components of high-quality healthcare delivery, directly influencing treatment adherence, health literacy, clinical outcomes, and patient satisfaction. Traditional patient education approaches often employ standardized materials and time-constrained verbal explanations that inadequately address individual patient needs, comprehension levels, or cultural contexts. ChatGPT has emerged as a potential tool to augment patient education delivery through accessible, interactive, and tailored information provision.

### A. Medical knowledge performance and educational content generation

Systematic evaluation of ChatGPT's medical knowledge through standardized examinations provides foundational evidence regarding content accuracy. Assessment using the United States Medical Licensing Examination (USMLE) demonstrated that ChatGPT-3.5 achieved 50%–60% accuracy,^17,18^ while GPT-4 showed substantial improvements, reaching 85%–92%, significantly exceeding passing standards.^20,212^ Evaluation using PubMedQA showed 78.2% accuracy when answering biomedical questions based on published literature.^213^ Performance on the Iranian medical licensing examination revealed 68.5% correct responses, substantially exceeding the 45% passing threshold and significantly outperforming random guessing, with superior performance in decision-making categories compared to basic science evaluation and diagnostics.^214^

Comparative analysis of AI-generated vs expert-authored patient education materials found that ChatGPT produced content with appropriate readability and clear organizational structure.^74,215^ However, systematic accuracy assessments identified occasional factual inaccuracies (5–20% error rates depending on complexity), omissions of clinically important information, and sometimes lacked technical precision present in professionally developed materials.^216,217^ Studies evaluating condition-specific education across cardiovascular disease, oncology, and diabetes found generally accurate fundamental information, though occasionally oversimplified complex concepts or provided incomplete treatment alternative discussions.^102,218^

### B. Discharge instructions and post-acute care

Emergency department discharge represents a critical transition point where inadequate understanding contributes to adverse events and preventable return visits.^219^ Studies examining ChatGPT's ability to enhance discharge instructions found AI-generated supplemental explanations improved patient comprehension, with significant improvements in correctly explaining diagnosis (78% vs 54%, p < 0.01), medication regimen (82% vs 61%, p < 0.01), and return precautions (85% vs 67%, p < 0.01) compared to standard instructions alone.^220,221^ However, instances where ChatGPT provided unnecessarily alarming information or inconsistent follow-up timelines highlighted the need for clinical review.^222^

Hospital discharge summary preparation showed a 60%–70% reduction in completion time when clinicians utilized ChatGPT for initial drafts, though requiring substantial physician editing (5–8 min average) for accuracy, completeness, and clinical nuance.^73,223^ In otorhinolaryngology clinical decision-making, ChatGPT achieved mean scores of 4.4/5, significantly lower than the specialist consensus of 4.91 (p < 0.001), with temporal instability evidenced by discordant responses in 25% of repeated queries.^224^

### C. Multilingual and cross-cultural communication

ChatGPT's multilingual capabilities spanning over 50 languages offer potential to enhance accessibility for non-English speaking patients.^57,225^ Studies evaluating performance across major languages, including Spanish, Mandarin Chinese, French, German, Arabic, and Hindi, found generally adequate translation quality and content accuracy, though with notable degradation for lower-resource languages.^35,226^ Comparative evaluation of Spanish-language patient education materials found equivalent or superior readability compared to professionally translated materials, with appropriate cultural adaptations.^227^ However, medical terminology accuracy varied, with occasional regionally inappropriate terms or literal translations lacking clinical precision, requiring native speaker review.^228^

### D. Medication education and adherence support

Medication non-adherence affects 30%–50% of patients with chronic conditions.^229,230^ Studies evaluating AI-powered medication education found improvements in patient knowledge, with significant increases in correctly explaining medication purpose (85% vs 62%, p < 0.001), identifying dosing schedule (91% vs 74%, p < 0.01), and recognizing serious side effects (78% vs 58%, p < 0.01) compared to standard counseling alone.^231,232^ However, direct evidence linking ChatGPT-delivered education to objectively measured adherence improvements through prescription refills or clinical outcomes remains limited.^233^

### E. Preoperative education

Evaluation of AI-generated materials for common surgical procedures found generally accurate procedural information and appropriate risk discussions, though with occasional omissions of procedure-specific details or generic recovery timelines not accounting for patient-specific factors.^234,235^

### F. Pediatric and adolescent health

ChatGPT can generate age-appropriate explanations, with studies finding generally positive adolescent attitudes, particularly for sensitive topics where anonymity was valued.^236,237^ However, concerns regarding sexual health information accuracy, mental health crisis response appropriateness, and potential normalization of risky behaviors require careful oversight.^238^

### G. Mental health psychoeducation

AI-generated mental health content showed generally accurate information with an appropriate empathetic tone.^239,240^ However, concerns include oversimplification risks, inability to assess suicide risk, potential to discourage professional treatment seeking, and lack of therapeutic relationship informing personalized recommendations.^241,242^ Clear boundaries, prominent crisis resources, and professional evaluation recommendations represent essential safeguards.^243^

### H. Preventive health

AI-generated recommendations showed appropriate guideline concordance for well-established preventive services from USPSTF and professional societies.^244,245^ However, accuracy declined for controversial recommendations, guideline disagreements, or interventions requiring shared decision-making incorporating patient values.^6^

## VIII. ChatGPT IN PREDICTIVE ANALYTICS AND DISEASE PREVENTION IN PRECISION MEDICINE

Predictive analytics underpins precision medicine by shifting healthcare from reactive treatment to proactive, individualized prevention. Integrating genomic, transcriptomic, proteomic, epigenetic, clinical, and lifestyle data enables early disease prediction and optimized health outcomes.^2,246^ Within this paradigm, AI-driven language models such as ChatGPT offer emerging capabilities to support predictive analytics and disease prevention.

ChatGPT, built on the Generative Pretrained Transformer (GPT) architecture, can process and synthesize large volumes of structured and unstructured biomedical data, ranging from EHRs and clinical narratives to omics and epidemiological datasets.^40^ While not designed for direct diagnosis, its strength lies in integrating heterogeneous data, detecting complex patterns, and generating interpretable insights for risk stratification and preventive decision-making (Fig. 9). As noted by Patrinos *et al.*, precision medicine increasingly depends on algorithmic models incorporating multi-omic and behavioral factors beyond population-level averages.^247^

### A. Predictive risk assessment and early detection

**FIG. 8.**
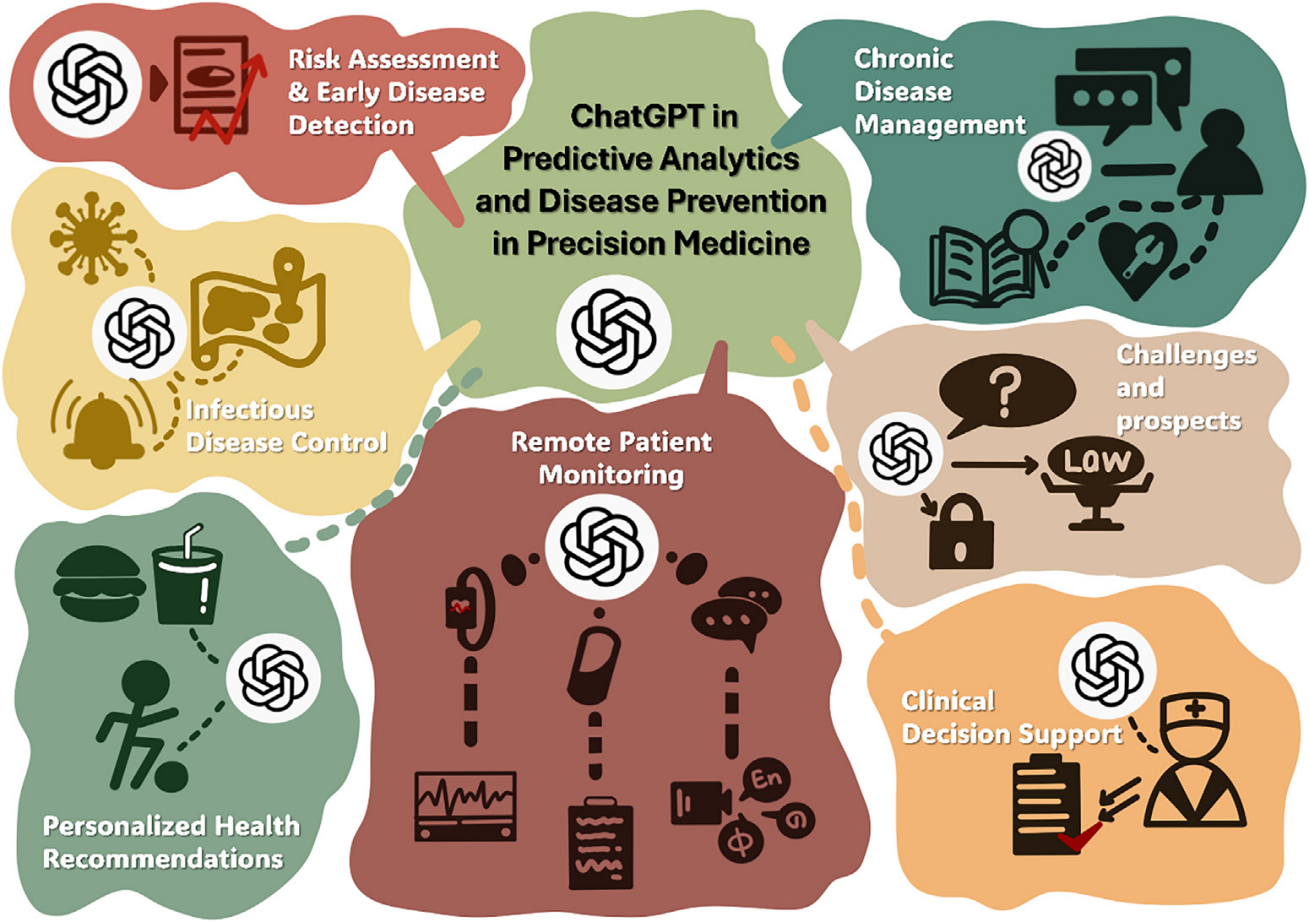
Detailed overview of ChatGPT in predictive analytics and disease prevention in precision medicine.

ChatGPT can enhance predictive risk assessment by synthesizing genetic, clinical, behavioral, and environmental data to identify individuals at heightened risk for complex diseases such as cardiovascular disorders, diabetes, cancer, and neurodegeneration (Fig. 8).^248,249^ The growing accessibility of multi-omic data and AI infrastructure has advanced disease prediction feasibility in routine care. As a decision-support interface, ChatGPT can translate intricate risk models into personalized narratives, advancing P4 medicine—predictive, preventive, personalized, and participatory by enabling early screening and tailored prevention strategies. Its interactive design may also strengthen patient engagement and shared decision-making.^250^

### B. Infectious disease surveillance and outbreak prediction

Beyond chronic diseases, ChatGPT can contribute to infectious disease surveillance by synthesizing diverse epidemiological signals from public health reports, scientific literature, and online data sources.^251^ AI-driven analytics, as demonstrated during COVID-19, enabled early outbreak detection and real-time monitoring. Integrated within surveillance systems, ChatGPT could enhance situation awareness, resource allocation, and rapid public health response (Fig. 8).

### C. Personalized prevention and chronic disease management

By integrating genetic risk, pharmacogenomic profiles, and behavioral data, ChatGPT can support individualized prevention plans, including lifestyle modifications, targeted screening, and tailored pharmacological strategies.^252^ In chronic disease management, it can aid in risk stratification, early intervention, and personalized care pathways, improving long-term outcomes. Its interpretive capacity may also enhance patient understanding and adherence to preventive regimens.

### D. Integration with remote monitoring and real-time analytics

The combination of ChatGPT with wearable sensors and remote monitoring devices enables dynamic, data-driven prevention. By interpreting longitudinal physiological data, ChatGPT can generate early alerts, summarize trends, and support clinician decision-making. Such integration reduces hospitalizations and enables timely intervention in chronic disease management (Fig. 8).^253^

Clinical Decision Support and Preventive Optimization: As a real-time decision-support tool, ChatGPT can synthesize guidelines, pharmacogenomic insights, and patient data to aid preventive care, identifying high-risk patients, predicting drug responses, and detecting potential interactions.^254,255^ However, human oversight is essential to safeguard accuracy, interpretability, and ethical standards, given persistent issues around bias, explainability, and regulatory compliance.

### E. Population-level prevention and public health surveillance

At a population scale, ChatGPT's analytical capability supports precision public health through integration of multi-source data to detect disease trends and inform preventive policy.^256^ These models enable the translation of individualized risk insights into broader health strategies.

## IX. ChatGPT IN REMOTE PATIENT MONITORING AND CHRONIC DISEASE MANAGEMENT

ChatGPT has emerged as a powerful tool in remote patient monitoring and chronic disease management, offering significant benefits to both patients and healthcare providers. By leveraging its advanced natural language processing capabilities, ChatGPT enhances various aspects of remote healthcare delivery and chronic condition management.

One of the key applications of ChatGPT in this domain is real-time patient data analysis. The AI can continuously monitor and interpret data from wearables, sensors, and other remote monitoring devices, providing timely insights into a patient's health status. This capability allows for early detection of health deterioration, enabling healthcare providers to intervene promptly and prevent potential complications.^257,258^ ChatGPT also facilitates personalized health management for patients with chronic conditions. It can offer tailored advice, medication reminders, and emotional support, helping to bridge the gap between clinical visits. This ongoing support is crucial for improving patient engagement and adherence to treatment plans.^259^ In the area of symptom monitoring, ChatGPT demonstrates significant utility. Patients or healthcare professionals can input symptoms and relevant information into ChatGPT-powered systems, which can then process and interpret this data. The AI can identify patterns, detect potential red flags, and alert healthcare if any concerning symptoms or changes occur. This proactive approach to symptom monitoring is particularly valuable in managing complex chronic conditions.^260^

Furthermore, ChatGPT enhances health literacy among patients with chronic illnesses. By providing easy-to-understand explanations of medical terminology and step-by-step guidance on medical instructions, ChatGPT empowers patients to take a more active role in their health management.^261^ In the context of mental health support, which is often a crucial component of chronic disease management, ChatGPT can provide conversational support and engage patients in therapeutic dialogues. It can even deliver guided cognitive behavioral therapy (CBT) exercises to help manage stress, anxiety, or depression associated with chronic conditions.^262,263^ ChatGPT also plays a significant role in enhancing telemedicine solutions. It enhances patient communication, offers multilingual support, and facilitates initial symptom assessments before patients meet with doctors. This integration can significantly improve the efficiency and accessibility of remote healthcare services.^264^

## X. OPPORTUNITIES AND CHALLENGES FOR ChatGPT IN PERSONALIZED MEDICINE

**FIG. 9.**
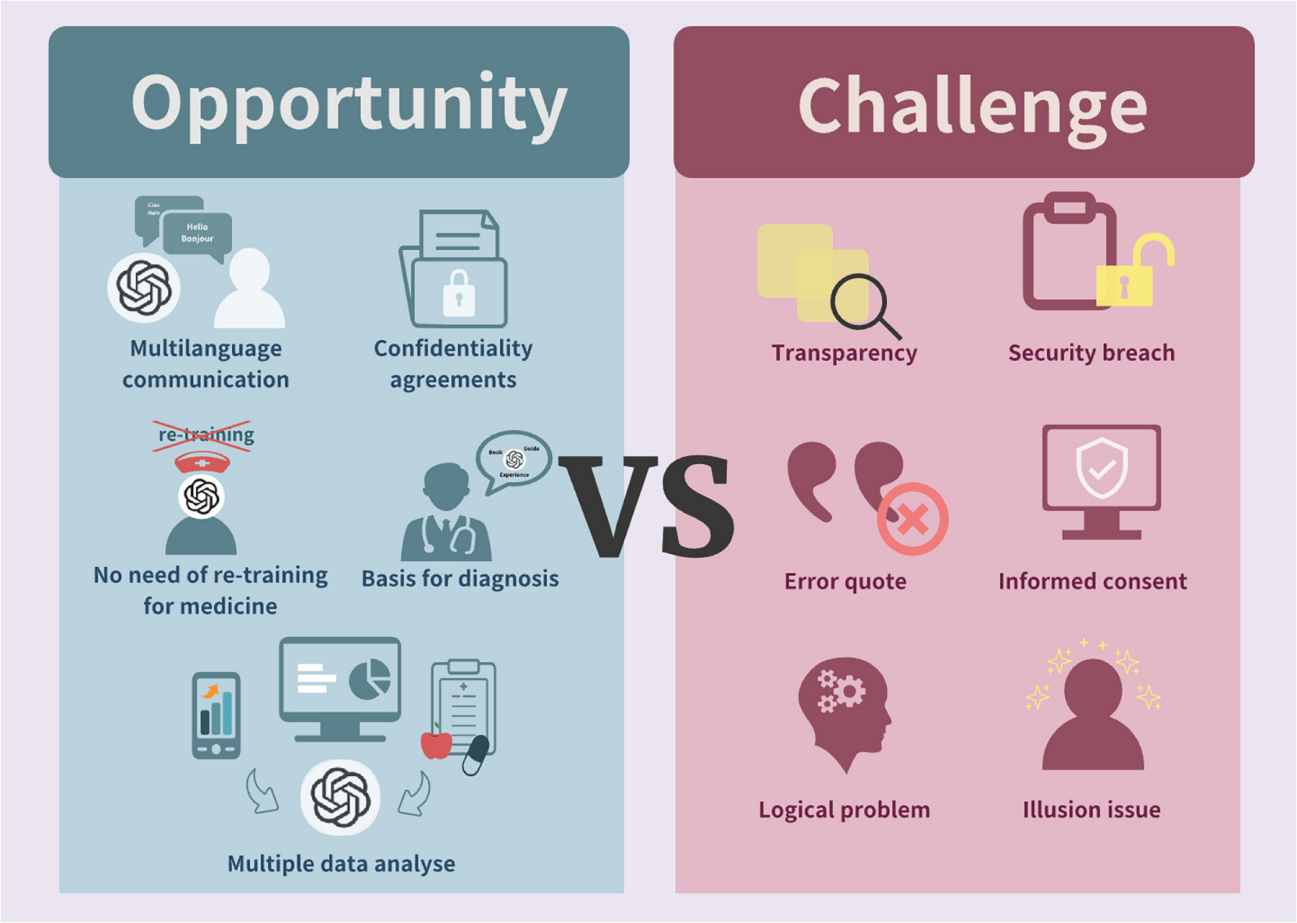
Overview of challenges and limitations facing ChatGPT.

ChatGPT presents both significant opportunities and challenges in the field of personalized medicine (Fig. 9). Its primary strength lies in its accessibility, facilitating efficient data collection, analysis, communication, and support across various fields of medical research.^265,266^ In medical research, ChatGPT shows promise in several areas, including drug discovery,^267^ diagnosis and treatment,^268^ personalized medicine,190 and patient monitoring. These advantages contribute to enhancing research quality, enabling proactive healthcare measures, and aiding disease outbreak prevention using predictive analytics.

Despite its potential, ChatGPT faces several challenges in the medical field. Ethical concerns, difficulties with regulatory compliance, and privacy and security issues are prominent.^269–271^ Ensuring the security of data storage and transfer protocols is crucial for safeguarding patient information. Additionally, bias in the training data can significantly affect research outcomes, thereby restricting ChatGPT's expertise in specific fields. The lack of personal interaction is another limitation, affecting data quality, especially in studies requiring interventions. Resource requirements pose short-term challenges, but integration with technical systems remains an issue.^264^

The use of ChatGPT for patient data raises ethical concerns, requiring robust protections. Mishandling could lead to severe consequences, necessitating encryption, HIPAA compliance, transparent communication, and constant validation. Continuous ethical oversight is vital in deploying AI in healthcare.^272^ Guidelines for reporting in medical research emphasize precise criteria definition, low-quality data assessment, and transparency about AI use. Standards advocate specifying hardware, software, and indications for use, integrating results into clinical treatment routes.^272^ Despite these challenges, ChatGPT positively impacts medical research by offering efficient access to medical information, personalized interventions, better data analysis, and improved cross-cultural communication in healthcare. Its integration into scientific research paves the way for new methodologies and better research outcomes.

## XI. FUTURE DIRECTIONS OF ChatGPT IN PERSONALIZED MEDICINE

Large language models (LLMs) such as ChatGPT have significant potential to advance personalized medicine by facilitating complex data integration, enhancing clinician-patient communication, and supporting longitudinal care.41 However, their safe and effective clinical application demands synchronized progress across technical innovation, clinical validation, regulatory compliance, and professional education. Future initiatives should therefore be conceptualized as an integrated roadmap rather than as independent technological advancements.

### A. Mitigating hallucinations via retrieval-grounded clinical reasoning

A major limitation of current LLMs is their tendency to generate plausible yet incorrect information, which poses unacceptable risks in clinical contexts.^23^ A practical mitigation strategy involves systematic adoption of retrieval-augmented generation (RAG), where outputs are anchored in authoritative sources such as clinical guidelines, pharmacological databases, genomic variant repositories, and institutional protocols.24 By constraining model responses to verified evidence and attributing explicit sources, RAG can reduce hallucinations, enhance trust, and enable clinician verification. Future models should benchmark hallucination frequency under RAG vs standalone generation as a key safety metric.

### B. Strengthening longitudinal context through memory-augmented architectures

Personalized medicine requires continuity across visits, comorbidities, and therapeutic adjustments, yet current models degrade over extended interactions. Effective strategies include memory-augmented and hierarchical attention frameworks combined with reinforcement learning from clinician feedback to emphasize clinically salient details over conversational noise.^273^ Evaluation paradigms must assess longitudinal reasoning through simulated multi-visit trajectories rather than single-turn accuracy alone.

### C. Multimodal integration with medical-grade evaluation

Next-generation ChatGPT systems should integrate textual, imaging, pathological, genomic, waveform, and wearable data to support holistic clinical reasoning.^274^ While multimodal foundation models enable cross-domain synthesis, deployment must follow rigorous medical validation, including modality-specific testing, subgroup analyses, and robustness assessments under real-world variability. Integration should be justified by improvements in diagnostic accuracy or decision quality, not technological novelty.

### D. Contextual domain adaptation for clinical deployment

Performance heterogeneity across specialties, rare conditions, and novel therapeutics necessitates focused domain adaptation. Ongoing pre-training and instruction tuning on specialty-specific corpora should directly align with defined clinical applications (e.g., oncology decision support, rare disease triage).^275^ Coupling domain adaptation with RAG ensures specialization enhances relevance without propagating outdated knowledge or bias.

### E. Privacy-preserving learning for multi-institutional evidence generation

Given the sensitivity of medical and genomic data, future development should leverage federated learning and differential privacy to enable multi-center improvement without data centralization.^216^ Research priorities include enhancing communication efficiency, managing institutional heterogeneity, and verifying resilience to inference attacks. These approaches are critical for producing generalizable, privacy-compliant evidence.

### F. Clinical validation and regulatory trajectories

Technical advancements alone are insufficient. Generative AI tools are increasingly classified as high-risk medical software, necessitating explicit intended use definitions, performance transparency, and post-market surveillance.^276^ Prospective clinical trials should assess not only accuracy but also impacts on patient outcomes, clinician workload, and decision quality relative to standard care. Continuous monitoring is essential to identify performance drift as guidelines and population characteristics evolve.

### G. Human–AI collaboration and workflow integration

Future systems must enable human-in-the-loop functionality, allowing clinicians to supervise, modify, and validate AI outputs.^277^ Providing uncertainty estimates, evidence provenance, and reasoning transparency will facilitate efficient review and uphold medicolegal accountability. Hybrid intelligence frameworks should be evaluated based on their ability to augment rather than replace clinical expertise.

### H. Medical education and workforce transformation

Sustainable integration of ChatGPT in personalized medicine demands transformation in medical education.^278^ Clinicians should be trained to critically interpret AI outputs, recognize biases and hallucinations, and employ AI effectively in shared decision-making. Curricular reforms should embed AI literacy, prompt engineering for clinical use, and ethical reasoning within evidence-based medical education.

### I. Ethical governance and equity-centered implementation

Finally, future research must embed ethical governance through equity audits, transparency mandates, and accountability mechanisms.^114^ Implementation science and health economic evaluation are essential to confirm that AI-enabled personalized medicine enhances health outcomes without deepening disparities or fragmenting care systems.

## XII. CONCLUSIONS

**FIG. 10.**
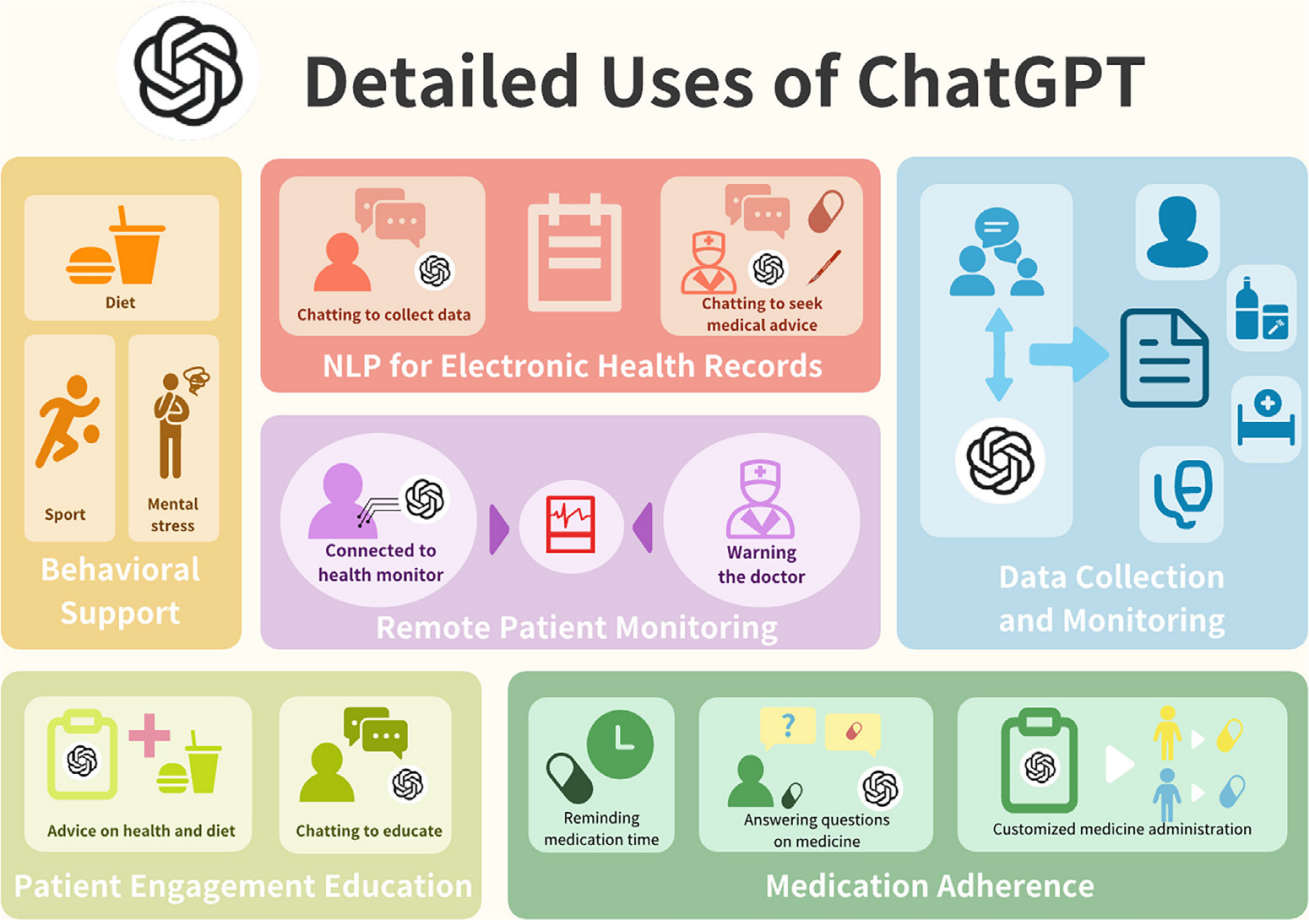
Detailed overview of ChatGPT's functionality and applications.

The current review elucidates the capabilities of ChatGPT in processing extensive datasets, deciphering complex contextual implications, and generating human like response (Table III). ChatGPT presents a promising avenue for advancing precision medicine, offering capabilities to enhance diagnostic and therapeutic efficacy across various domains (Fig. 10). Clinical investigation of ChatGPT can aid clinicians and researchers to better understand the pathophysiology of diseases such as cancer, cardiovascular and cerebrovascular disorders, HIV/AIDS, and diabetes. The capacity to analyze large-scale genomics and clinical datasets, coupled with its natural language processing abilities, positions it as a valuable tool for personalized risk assessment planning. In conclusion, while further refinement and validation are needed, ChatGPT offers a significant opportunity to advance personalized and precise medical practices, ultimately improving patient outcomes and healthcare efficiency.

**TABLE III.** Performance evaluation metrics for ChatGPT in precision healthcare.

Metric	Category	Definition	Challenges Addressed	Performance Benchmark	References
Diagnostic Accuracy	Clinical Performance	Measures the correctness of diagnoses and clinical decision-making	Misdiagnosis, irrelevant responses	72% overall accuracy in clinical decision-making	70
Final Diagnosis Accuracy	Clinical Performance	Assesses accuracy in making a final diagnosis	Incorrect final diagnoses	77% accuracy	70
Differential Diagnosis Accuracy	Clinical Performance	Evaluates the ability to provide a set of possible diagnoses	Incomplete or inaccurate differential diagnoses	60% accuracy	70
Clinical Management Accuracy	Clinical Performance	Measures accuracy in making care management decisions	Inappropriate treatment or management plans	68% accuracy	70
Medical Information Accuracy	Content Quality	Evaluates the correctness of medical information provided	Misinformation, outdated knowledge	Median accuracy score of 5 out of 6	70
Completeness	Content Quality	Assesses comprehensiveness of answers to medical queries	Incomplete or partial information	Median completeness score of 3 out of 3	113
Adherence to Simulation Parameters	Clinical Performance	Measures the ability to follow basic and advanced simulation parameters	Unrealistic or inconsistent patient scenarios	100% adherence to basic parameters, 55% success in delaying feedback	279
Empathy	User-Centered	Evaluates emotional sensitivity and engagement in patient interactions	Lack of personalization and emotional support	70–80%	280
Trustworthiness	Safety and Security	Ensures ethical compliance and avoidance of toxicity in responses	Ethical concerns, potentially harmful advice	90% decrease in harmful content	281
Latency	Computing Performance	Measures response time for generating and delivering prompts	Delayed communication during patient interactions	2–3 s	282

## Data Availability

The data that support the findings of this study are available within the article.
